# Distance Learning During the First Lockdown: Impact on the Family and Its Effect on Students' Engagement

**DOI:** 10.3389/fpsyg.2021.762213

**Published:** 2021-11-11

**Authors:** Antonella Chifari, Mario Allegra, Vincenza Benigno, Giovanni Caruso, Giovanni Fulantelli, Manuel Gentile, Lucia Ferlino

**Affiliations:** Institute for Educational Technology, National Research Council of Italy, Genova, Palermo, Italy

**Keywords:** emergency remote education (ERE), students' engagement, COVID-19, family involvement in education, home learning environment (HLE)

## Abstract

This contribute investigates how Emergency Remote Education (ERE) impacted families during the spring 2020 Covid-19 lockdown, and in particular, the extent to which the impact of ERE on families, measured in terms of space and equipment sharing, moderates the effect of student and family characteristics on students' engagement. The study derived from the administration of an online survey to 19,527 families with children attending schools, from nursery to upper secondary grade. The total number of student records collected amounted to 31,805, since parents had to provide data for each school-age child in the family. The survey contains 58 questions, divided into three sections, with the first two sections designed to get a reading at family level and the third section to gather data for each school-age child in the family. After verifying the validity of the engagement construct through confirmatory factor analysis, two structural equation models were used to analyze the students' engagement. The main findings reveal how the impact of the ERE on the families has had a significant role in predicting students' level of engagement observed by parents with respect to different predictor variables. Finally, we argue that it is necessary to follow a holistic approach to observe the challenges imposed by the switch of the process of deferring teaching from presence to distance, imposed by the pandemic emergency on families. In fact, a holistic approach can promote student engagement and prevent the onset of cognitive-behavioral and affective problems linked to disengagement in ERE.

## 1. Introduction

The onset of the Covid-19 pandemic in the early 2020s forced most educational institutions to suspend face-to-face teaching activities and move toward distance learning to contain the spread of the epidemic. Not all European countries have adopted the same measures, some have progressively readmitted students to school after the most critical period, others, like Italy, implemented nationwide school closures as of 9 March 2020 until the end of the school year (United Nations Educational, 2021). The closure of school in presence on our national territory was firstly established with a Decree by the Prime Minister (DPCM 4 March 2020), and confirmed with subsequent similar decrees (DPCM of 26 April 2020; D.L. 16 May 2020, n. 33; DPCM 17 May 2020); teaching was provided online until the end of the school year. Therefore, Emergency Remote Education (ERE) represented a temporary solution, the only way “to survive in a time of crisis with all resources available, including offline and/or online” (Bozkurt and Sharma, 2020, p.2), realistically a branch of online learning and homeschooling.

It is interesting to note that during the period of school closures caused by the pandemic, the focus of many studies has been on what happens when the classroom space-time setting moves into the home environment, introducing the multi-faceted world of learning in the digital age into the rhythms of family life (Benigno et al., 2021; Gentile et al., 2021). Scholars and academics worldwide have searched answers to the many research questions raised by the ERE, mainly from the schools' and teachers' perspective: how schools and teachers managed the emergency.

However, the number of scientific papers on how students reacted, in terms of student engagement, to ERE is still very deficient. In particular, there is a gap in the literature concerning the study of engagement in relation to its impact on the cognitive-behavioral and affective attitudes toward the new online learning experiences during the pandemic crisis. Starting from this, the motivation for the study presented in this paper is to analyze the impact of ERE on students' engagement — defined in terms of cognitive-behavioral and affective attitudes toward ERE — considering at the same time both the support provided by the Italian families during the homeschooling period and the educational approach followed by teachers.

The importance of considering the complexity and diversity of families' settings to cope with ERE from home has been highlighted by several studies (Brom et al., 2020; Di Pietro et al., 2020; Hapsari et al., 2020; Pellegrini and Maltinti, 2020), which have investigated specific variables influencing ERE activities (e.g., the number of children engaged in ERE at the same time; the number of parents working from home, at full or part time; availability of separate space for studying or the necessity to share house spaces; availability of technology equipment in the household). It is reasonable to assume that comfortable and arranged family conditions, with the necessary space to work and technological equipment, foster a more positive and productive engagement for studying and, more in detail, to complete the assigned learning tasks. Unfortunately, this might not be the case for many students who live in a disadvantaged condition, in which they carry out their work in a small space shared with other family members. Several studies reveal considerable differences in housing quality across European countries, and capture important disparities that affect children's engagement and goals achievement (Di Pietro et al., 2020; Gigli, 2020; Ndhine, 2020).

Besides, it is important to reflect non only on the different families' conditions, but also on the educational challenges raised by the ERE context. According to Affouneh et al. (2020), the course design, assessment, and teaching strategies originally planned for face-to-face teaching have changed. Teachers, students, and parents have had to adapt very quickly to new educational scenarios where the use of technology plays a very strong and important role. The massive and not always prepared use of emerging technologies, during the crisis, has reshaped different learning aspects, influencing the student's engagement (Bergdahl et al., 2020; Khlaif et al., 2021), and impacting on their affective, cognitive and behavioral attitudes toward the educational experiences. Theoretically, student engagement is defined as “the student's psychological investment in and effort directed toward learning, understanding, or mastering the knowledge, skills, or crafts that academic work is intended to promote” (Newmann et al., 1992, p.12).

It plays a crucial role in students' learning and satisfaction in distance education because online learners seem to have fewer opportunities to be engaged with positive learner experiences and interactions with content, peers, and instructors (Bolliger and Martin, 2018; Martin and Bolliger, 2018).

However, if before COVID-19 several studies have focused on online learning trying to identify the factors influencing student's engagement in normal situations (Fugate et al., 2018; Wong and Chong, 2018), there is a lack of research about the distinct components influencing student's engagement in online learning during the COVID-19 pandemic emergency. So, in accordance with Bond's definition of engagement, that is rooted in the communities of learning paradigm, engagement represents “the energy and effort that students employ within their learning community, observable via any number of behavioral, cognitive or affective indicators across a continuum” (Bond and Bedenlier, 2019, p.3). Following this definition, the present study explores the construct of engagement taking into consideration both the cognitive-behavioral and emotive-affective factors which influence student engagement in the Italian families, during the ERE in the period of COVID-19 crisis.

Consequently, through the administration of an online survey to a representative sample of 19,527 Italian families, the current study tries to answer the following research questions:

- Q1 How do specific ERE strategies adopted by teachers directly impact on students' engagement?
- Q2 How do specific students characteristics directly impact on students' engagement?
- Q3 How do specific family characteristics directly impact on students' engagement?
- Q4 How does the impact of ERE on the family moderates the influence of students and family characteristics on students' engagement?

The choice of the variables to be investigated, both those related to the student characteristics and those related to the family characteristics and the educational context, has guided the entire research process. A process based on a systemic approach according to which studying the construct of engagement could not be separated from the analysis of what could influence it, directly or indirectly. Thus, it became very important to understand how, during the pandemic period, variables related to the family or the instructional contexts could provide sensible and practical answers to why student engagement was not always constant, but often depending on the situational antecedents that trigger it and the consequences that maintained it.

Studying the strength of this impact, in such a large sample, will add an important piece of knowledge to the current literature on engagement by relating it not only to the quality of the learning provided, but also to the quality of the family support given to the students who had to face the challenge of emergency remote education.

## 2. Method

### 2.1. Participants

The survey participants were 19,527 families, and the total number of child records collected amounted to 31,805. This difference is due to the design choice of allowing parents to provide data for each of the school-age children in the family, thus obtaining a more detailed picture of the impact of distance learning. The responding parent was generally the mother(86,7%). Considering data from all parents (both respondent and other parents), 30.8% of them are between 45 and 49 years old; 94.4% of them are Italian citizens. About the level of education, 36.5% of parents have a university degree, and 46.2% have a secondary school diploma. In terms of employment status, 77.2% of parents were employed, and 10.6% were unemployed. Data reveals that 55.3% of the parents interviewed worked from home during the lockdown period. The sampled families live prevalently in the regions of Central Italy (68.9%). The sample of children includes kids in nursery school (10.2%), and pupils at first cycle primary schools (16.6%), second cycle primary schools (23.3%), first cycle secondary schools (25.5%) and second cycle secondary schools (24.5%). The presence of a disability was reported for 905 children out of 31805. These students are distributed among the following school levels: 11.2% preschool, 21.7% first-cycle elementary, 20.3% second-cycle elementary, 26.3% first-cycle secondary, and 20.6% second-cycle secondary.

### 2.2. Design

The objective of the survey was to obtain a comprehensive picture of the impact of distance learning on the families during the Covid-19 emergency. To this aim, during the first phase of the COVID-19 pandemic (March-May 2020), we structured the questionnaire in such a way to allow parents to report data for each of the school-age children in the household. It contains 58 questions, divided into three main sections, with the first two sections designed to gather information regarding the family as a whole, and the third section to collect data for each child in the family. Considering the use of the survey as a tool to investigate the impact of ERE on families, the present study complies with the approach provided by selective studies (Kish, 1987; Ato et al., 2013).

### 2.3. Procedure

The questionnaire was administered online through the open-source software LimeSurvey, and spread through the snowball sampling technique between May 12 and June 22, 2020. The data collection has been conducted according to the regulation established by the General Data Protection Regulation (GDPR, 2016). In particular, the questionnaire has been designed according to the privacy by default principles, as specified in the article 25 of the Regulation, so as to reduce the personal and special data to be collected, and minimize the ethical impact as indicated by Hoerger and Currell (2012). Specifically, all the collected data are anonymous, thus minimizing the risk that this information could lead back to the identity of the participants. Furthermore, the LimeSurvey software used to administrate the questionnaire has been installed on the Institute's management server in order to maintain total control over the life cycle of the data and the technical information collected by the servers themselves and necessary for the use of the questionnaire (e.g., IP address, type of browser, etc.). Finally, along with the questionnaire we provided an informative letter containing details on the purpose of the research, the authors, and any other information useful for understanding the scientific context in which the survey has been conducted.

### 2.4. Instrument

The first set of questions concerns the socio-demographic profile of the family. In particular, parents' personal data (gender, age, nationality, citizenship, school level, employment status), and general data on the composition of the household (i.e., the number of adults and number of children of school age) have been collected. In this first section, we investigated whether one or both parents worked from home (WFH) during the pandemic and if they needed support to manage their child(ren) (whether they worked from home or not). The second section provides a picture of families' technological endowment and their initial capacity to respond to distance learning and parents' WFH requirements. In particular, whether or not they had immediate availability of the equipment necessary to attend remotely, and whether they were in some way “forced” to equip themselves independently or with the support of the school. Resources availability and parental confidence in information technology constitute an essential part of the questionnaire useful in corroborating the studies on socio-cultural and instrumental inequalities highlighted by many of the previously mentioned studies. The third section of the questionnaire collects information about the family's distance learning experience concerning the individual child. Therefore, parents fill out a form for each school-age child, specifying the school leveland the presence of any disabilities or special educational needs. This section investigates the impact that distance learning has had on family management, whether it has had repercussions on daily life and shared spaces organization. An additional set of questions was proposed to detect families' perceptions related to the educational effectiveness of distance learning and school organization, both in terms of support and communication with the families. Specific questions were also formulated to understand whether their children's psycho-physical well-being was affected during the lockdown period and whether noteworthy changes were observed in the socio-affective and behavioral domains. Finally, in the case of a child with disabilities parents were asked to report how distance learning had ensured inclusion even in the virtual context.

### 2.5. Statistical Analysis

To analyze students' engagements during ERE, we focused on the responses provided by parents to the question “What attitude do you notice in your son/daughter toward distance education?”. In particular, through this question, we asked parents to what extent (in a scale from 0 to 10) they had noticed the following attitudes: cooperation, curiosity, interest, concern, restlessness, and emotional volatility.

First, we conducted a qualitative investigation calculating the descriptive statistics on the scores for these items. Then we analyzed these attitudes observed by the parents according to the engagement model reported by Bond and Bedenlier (2019) and Bond (2020a,b), where the construct of engagement is defined as a second-order latent variable built on the first-level latent variables (affective and cognitive-behavioral). Specifically to the *Affective* component, a less restless, worried and volatile behavior observed in the student corresponds to greater involvement in distance learning activities. Similarly, concerning the *Cognitive-behavioral* component, the level of engagement increases when their family members perceive students as very interested, collaborative and curious about learning. We verified the factorial structure of the engagement model through a Confirmatory factor analysis (CFA).

Next, we studied student engagement as certain conditions varied. In particular, we considered the following variables grouped in three areas:

- the family context
  - age of the parents, calculated as the maximum age of the two parents
  - school level of the parents, calculated as the maximum school level of the two parents
  - residence, considered ad the macro-area of Italy (North, Center and South Italy)
  - citizenship
  - presence of at least one parent in remote working
- the characteristics of the student
  - gender
  - school level
  - presence of disabilities
- the teaching approach used during the ERE
  - technology tools used during ERE
  - implementation of collaborative activities during ERE.

We verified the effects of these variables on the students' engagement by means of a structural equation model (Model A) (Q1,Q2,Q3).

Finally, we fitted a second structural equation model (Model B) to check if and how the impact of ERE on the family moderates the effect of the variables related to family background and student characteristics over the students' engagement (Q4). The impact of ERE on the family was defined as a latent variable observed by the responses of two items through which we asked parents about the impact of distance education on space sharing and instrumentation sharing.

Both the models was defined as multilevel structural equation model to cope with the hierarchical structure of the collected data. In fact, allowing the parents to provide data for each school-age child we obtained a sample of 19,527 families and 31,805 students; indicatively, an average of 1,63 student records was reported for each family. Data about students represent the first-level units, while the second-level units are the family.

For the purposes of this article of the 31,805 student records collected, the 25,563 student records for which distance education was enabled were analyzed. Structural models were estimated on the 20,586 student records for which the variables investigated were found to have no null values.

Multiple fit indices were considered to check the models : the root mean square error of approximation (RMSEA), the standardized root mean squared residual (SRMR), and the comparative fit index (CFI). RMSEA levels of < 0.05 indicate a good fit while values < 0.08 indicate an acceptable fit (Kline, 2015). SRMR < 0.05 represents a good fit and < 0.10 is acceptable (Schermelleh-Engel et al., 2003). Finally, CFI values of > 0.97 can be considered a good fit (Schermelleh-Engel et al., 2003), and > 0.95 can be considered an acceptable fit (Schermelleh-Engel et al., 2003).

All the analyses on student-level variables were carried out using the Lavaan package (Rosseel, 2012) of the open-source software R (R Core Team, 2018).

## 3. Results

The 20,586 student records used to estimate the structural model are divided into 10,497 records referring to male students and 10,089 to female students. In 570 cases, parents reported the presence of disabilities. The 20,586 cards are composed by 3,476 cases relating to the first cycle of the primary school, 5,050 to the second cycle of the primary school, 5,985 to students of the first degree secondary school and 6,075 to students of the second degree secondary school. In the structural models, a variable representing the linear component of a polynomial contrast matrix was used to assess the effects on engagement as the school level increases. In 8,831 out of 20,586 cases, at least one parent in remote working was present in the household. Concerning citizenship, only in 364 cases did the respondent household declare itself a first or second-generation immigrant. The vast majority of the responding households were residents in central Italy (14,020), while 5,027 and 1,539 households were residents in northern and southern Italy. The parents' educational level shows a prevalence of families with a university degree (10,598) or a high school diploma (10,479). In 3,233 cases, parents declare a Bachelor degree, while there are residual cases of parents with a secondary school license (1,232), an elementary school license (11) or no qualification at all (10). As in the case of students' school level, it was chosen to use a variable capable of representing the linear component of this ordinal variable. Table 1 shows the distribution of the age of the parents.

**Table 1:** Distribution of parents' age.

**Age**	***n***
<20	8
20–24	3
25–29	21
30–34	192
35–39	1, 017
40–44	3, 890
45–49	6, 965
50–54	5, 886
>54	2, 604

Table 2 shows the frequencies of technologies adoption during ERE.

**Table 2:** Frequencies of technologies adoption during ERE.

**Variable**	***n***	**Freq**
Collaborative activities	8,073	0.39
Video conference systems	16,925	0.82
Online learning platform	10,887	0.53
Shared folders	3,012	0.15
Publisher learning resources	1,119	0.05
Electronic registry	15,737	0.76
Messaging	8,502	0.41

Mean scores, standard deviations, together with skewness and kurtosis for the investigated observed students' behaviors are shown in Table 3.

**Table 3:** Descriptive statistics of observed students' behaviors.

	**Variable**	**Mean**	**SD**	**Skewness**	**Kurtosis**	**% Missing**
1	Collaborative	6.89	2.55	−0.95	0.41	0
2	Curious	6.47	2.56	−0.79	0.13	0
3	Emotionally volatile	3.75	3.45	0.35	−1.28	0
4	Interested	5.46	2.75	−0.42	−0.55	0
5	Restless	3.81	3.36	0.33	−1.21	0
6	Worried	4.03	3.53	0.23	−1.36	0

According to the Kolmogorov-Smirnov test, all items show significantly non-normal distributions (*p* < 0.0001 for all the items).

Table 4 shows the correlations between individual attitudes highlighting the significant correlations, with an absolute value ranging between 0.14 and 0.86. Particularly strong are the correlations between the collaborative, curious, and interested items and between the restless, emotionally volatile, and worried items.

**Table 4:** Correlations of observed students' behaviors.

	**Collaborative**	**Curious**	**Interested**	**Restless**	**Emotionally volatile**
Collaborative					
curious	0.86^****^				
Interested	0.62^****^	0.73^****^			
Restless	–0.14^****^	–0.16^****^	–0.12^****^		
Emotionally	–0.41^****^	–0.42^****^	–0.33^****^	0.54^****^	
volatile					
Worried	–0.36^****^	–0.38^****^	–0.29^****^	0.52^****^	0.76^****^

**** *p < 0.0001*.

**Figure 1:**
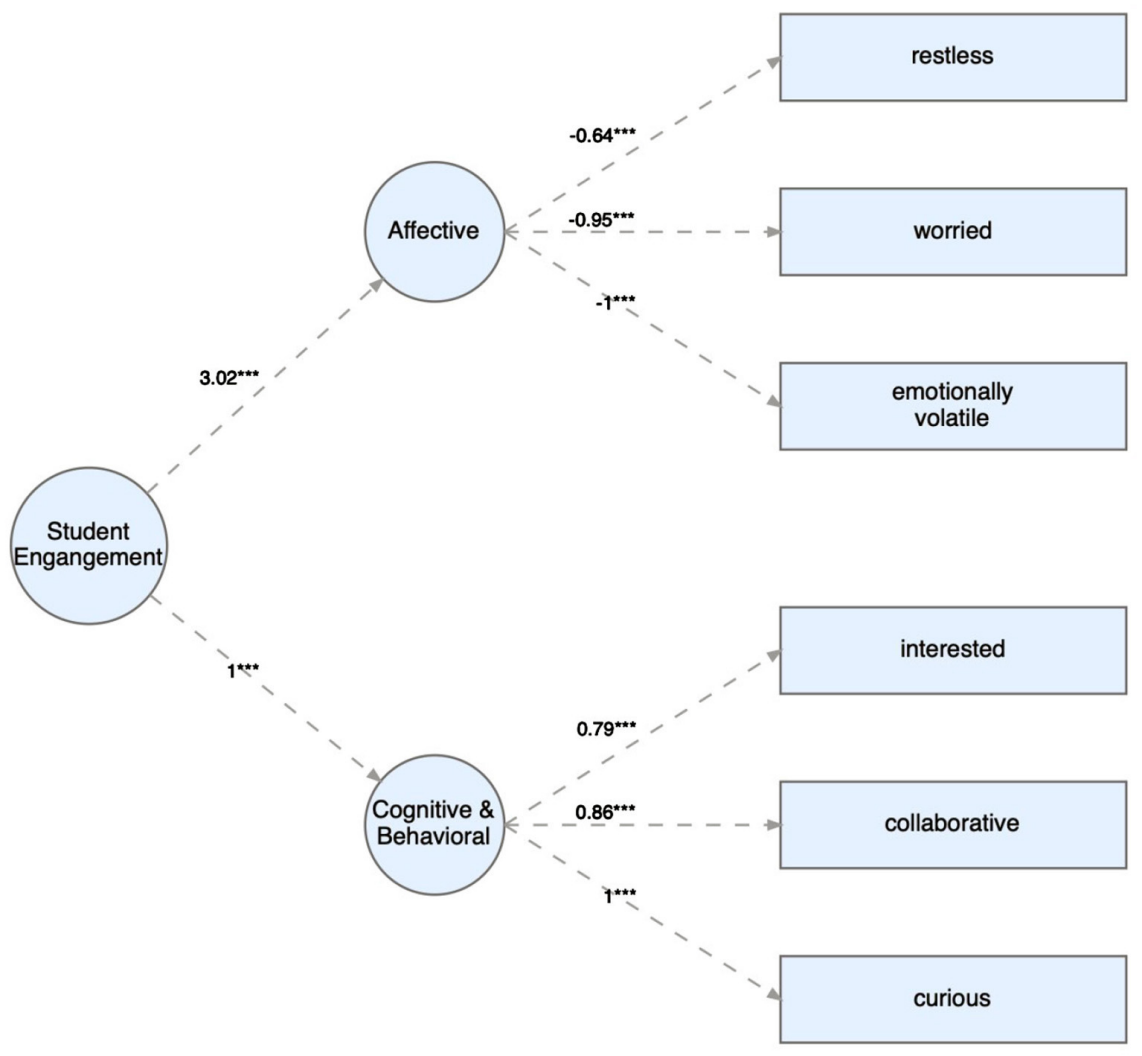
CFA students' engagement. ***p < 0.001.

The CFA of the engagement construct built on observed behaviors in accordance with Bond and Bedenlier (2019) and Bond (2020a,b) shows a good fit according to all the fit indices (*SRMR* = 0.03, *CFI* = 0.99) except for RMSEA (*RMSEA* = 0.08). Figure 1 shows the factor loadings of the engagement model.

The model A shows a good fit according all the fit indices (*RMSEA* = 0.04, *SRMR* = 0.02, *CFI* = 0.96). Also, model B shows a good fit according all the fit indices (*RMSEA* = 0.04, *SRMR* = 0.02, *CFI* = 0.96).

**Figure 2:**
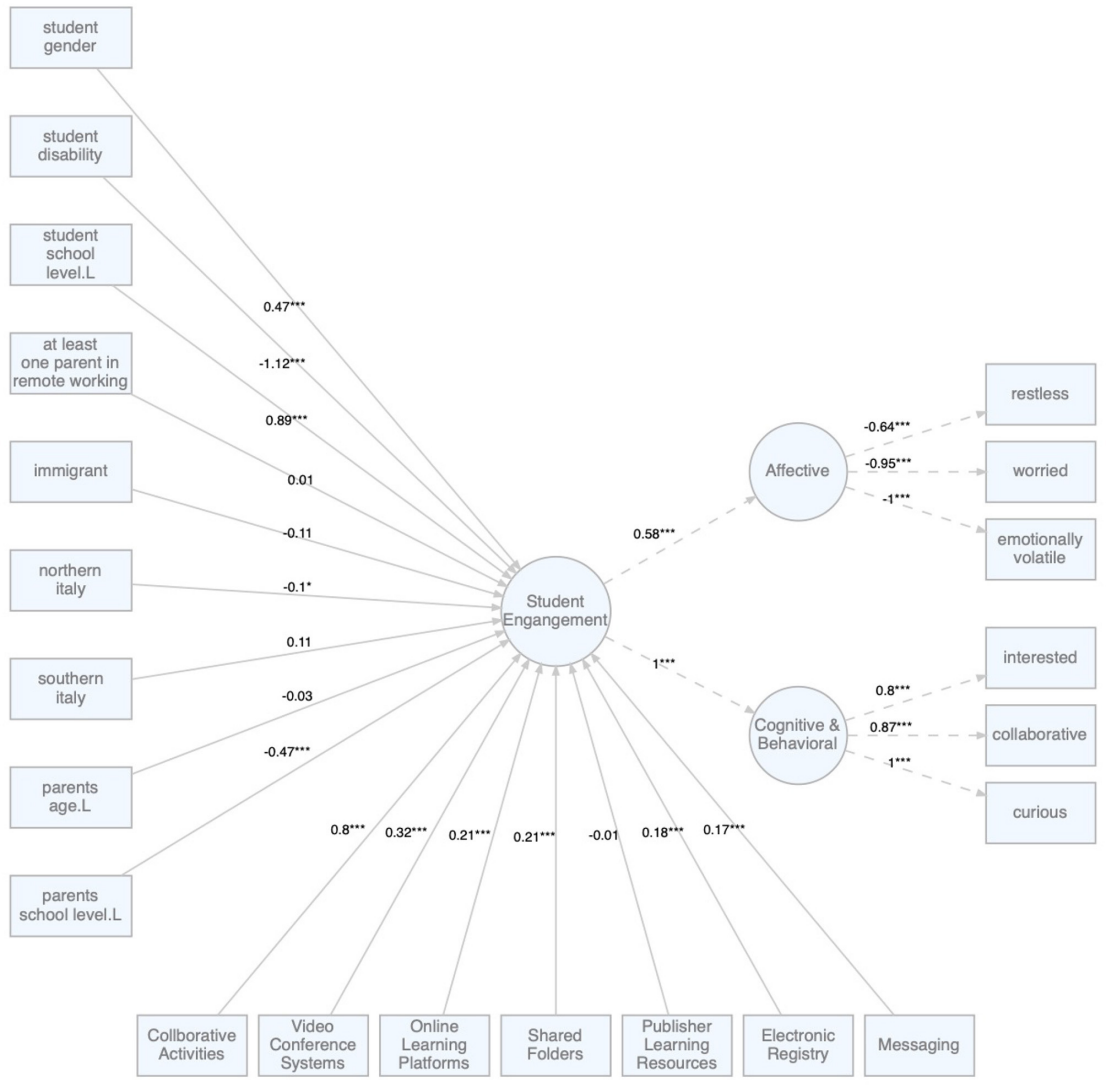
Model A: Effects of student and family characteristics on students' engagement. *p < 0.05; ***p < 0.001.

**Figure 3:**
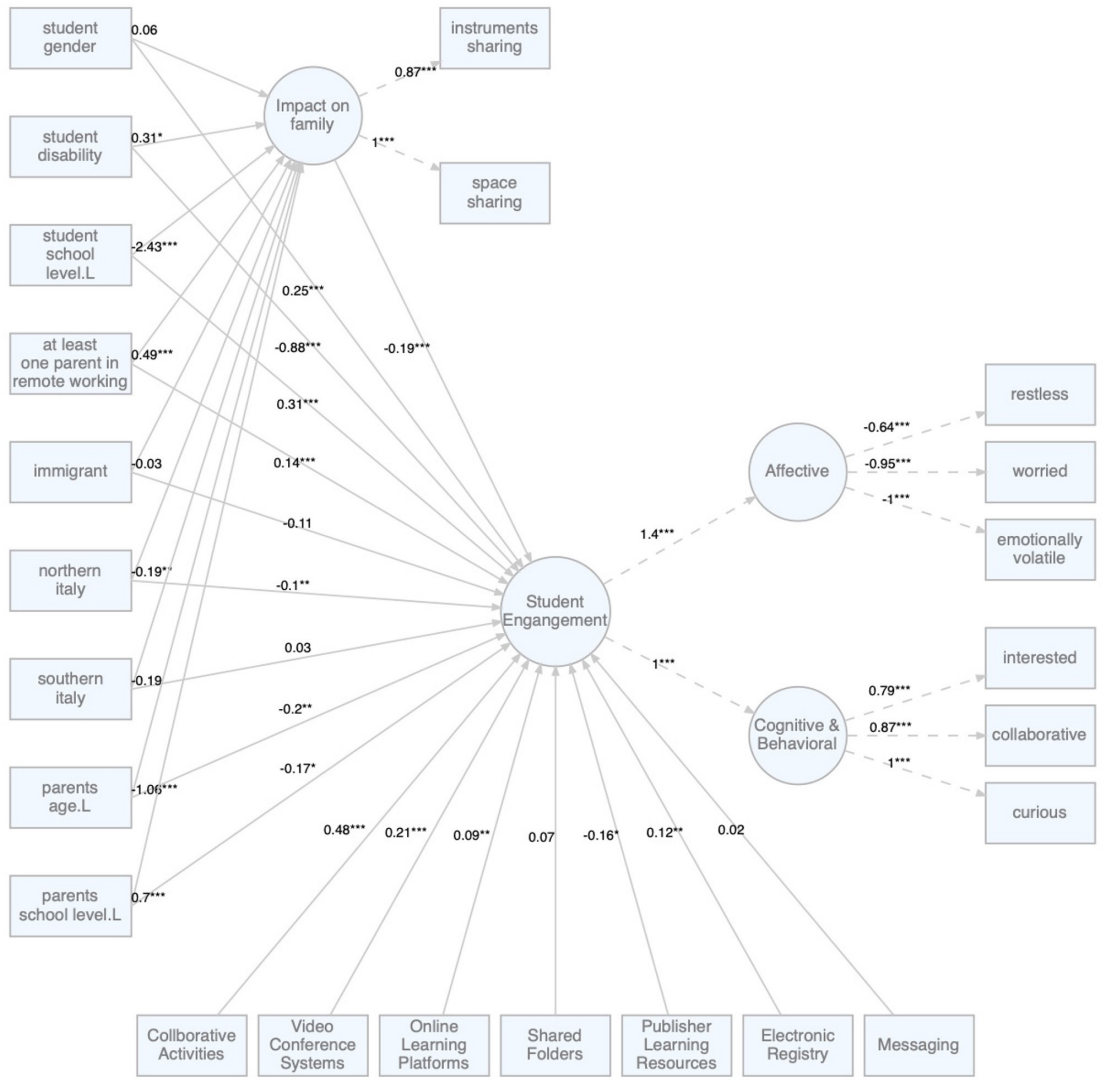
Model B: The role of distance learning family's impact on students' engagement. *p < 0.05; **p < 0.01; ***p < 0.001.

Figures 2, 3 report the factor loadings and the regression coefficients of the two structural models.

Table 5 shows the estimates of indirects effects, respectively on the engagement latent variable, mentre la Table 6 shows the estimates of total effects of the variable on the engagement latent variable.

**Table 5:** Estimates of indirect effects on engagement.

**Label**	**EST**	**SE**	***z***	***p*-value**	**CI.lower**	**CI.upper**
ab_s_gender_impact	−0.01	0.01	−1.23	0.218	−0.03	0.01
ab_s_disability_impact	−0.06	0.03	−2.19	0.029	−0.11	−0.01
ab_s_school_level_L_impact	0.46	0.02	22.69	0.000	0.42	0.50
ab_p_SW_impact	−0.09	0.01	−7.84	0.000	−0.11	−0.07
ab_p_immigrant_impact	0.01	0.04	0.14	0.891	−0.07	0.08
ab_p_northern_italy_impact	0.04	0.01	2.92	0.003	0.01	0.06
ab_p_southern_italy_impact	0.04	0.02	1.71	0.088	−0.01	0.08
ab_p_age_L_impact	0.20	0.02	9.73	0.000	0.16	0.24
ab_p_school_level_L_impact	−0.13	0.02	−5.37	0.000	−0.18	−0.08

**Table 6:** Estimates of total effects on engagement.

**Label**	**EST**	**SE**	***z***	***p*-value**	**CI.lower**	**CI.upper**
total_s_gender	0.24	0.04	6.19	0.000	0.16	0.31
total_s_disability	−0.94	0.11	−8.37	0.000	−1.16	−0.72
total_s_school_level_L	0.76	0.06	12.42	0.000	0.64	0.89
total_p_SW_impact	0.05	0.04	1.35	0.177	−0.02	0.12
total_p_immigrant_impact	−0.10	0.13	−0.81	0.417	−0.36	0.15
total_p_northern_italy_impact	−0.06	0.04	−1.66	0.097	−0.14	0.01
total_p_southern_italy_impact	0.06	0.06	1.02	0.310	−0.06	0.19
total_p_age_L_impact	0.00	0.06	−0.01	0.989	−0.12	0.12
total_p_school_level_L_impact	−0.30	0.08	−3.83	0.000	−0.45	−0.15

## 4. Discussion

The results analyzed according to the two structural models described above and obtained through the wide survey carried out on Italian families during the period of the first lockdown (March-June 2020), explain how some variables related to the family context, to the student's characteristics and to the didactic approach influenced the families' perception of their children's emotional-affective and cognitive-behavioral involvement during the remote education activities.

The first of the two models used (Figure 2) highlights the effect of some descriptive variables on students' engagement. In particular, the model analyzes how some characteristics of the family (parents working from home, migrant family, area of residence, parents' age and school level), of the student (gender, disability and school level) and of the remoteness education approach adopted during the pandemic emergency influenced students' engagement.

Specifically to the educational approach, the model shows that parent's perception of children's engagement is higher especially where collaborative activities were proposed, thus confirming the findings by Bolliger and Martin (2018). The use of videoconferencing systems, online platforms and shared folders have played a key role in keeping interest and motivation high. The effect of using these tools have resulted in more effective motivation than using other online tools. Therefore, we can deduce that the education approach used during the remote activities has had a strong impact on the children's engagement, especially when it has adopted teaching strategies and support tools which are contextually appropriate and motivationally salient.

These findings support the idea that, in order to be effective in engaging the student's interest, ERE must have characteristics that make it not a mere transposition of face-to-face teaching, but rather a corpus of activities properly designed for the distance setting (Zhu et al., 2016). Furthermore, ERE should provide students with rich, holistic learning opportunities during the pandemic lockdown engaged relatively frequently and successfully with online learning (Domina et al., 2021).

Specifically to the students' characteristics that influenced her/his engagement model A has highlighted that the gender of the students had a significant impact on engagement; in particular, female students were more involved than their male peers. This result is confirmed by a number of studies showing that female adolescent students can achieve significantly more positive online learning outcomes than males because they are more persistent, motivated, and more self-regulated than their male peers (Yu, 2021). In particular, Korlat et al. (2021) in their study examining the relationship between gender and level of engagement, state that if boys are perceived as more able than girls in online activities, girls are more engaged in learning activities and more study oriented. Thus, it could be that girls are more likely to have transferred their previously established learning practices to the new online learning context. Similarly, in Hsiao (2021) study, the male gender tends to be more passive in learning and consequently boys' independent learning ability may require further improvement.

Individuals with disabilities are perceived by their parents to be less involved (β = −1.12**), in addition, the analysis reported in Model B confirms both a direct effect on engagement (β = −0.88***) and an indirect effect through an increased impact on families (β = 0.31*). We hypothesized that children with disabilities, during the period of the pandemic emergency, as Parmigiani et al. (2020) suggests, suffered more from the loss of contact with their peers and teachers, thus making the family feel less involved in their studies and more socially isolated. In addition, their presence at home, being less autonomous and deprived of the support they usually enjoy in class, had a greater impact on the family who had to, often without external help and very quickly, provide their own care and share both physical and temporal spaces.

Besides, we note that there is a small difference between central and northern Italy and that, in particular, students from northern Italy seem to have been less involved than those from central Italy, while there are no significant differences between the south and the center. It is remarkable to point out that when the school level of the students grows, engagement increases, and that parents' age and working from home do not impact on the perception of parents' level of engagement. Similarly, no significant differences have emerged between families with migrant backgrounds and native families.

The parent's school level, on the other hand, has a significant impact in the sense that parents with high schooling seem to perceive lower engagement of their children. This is probably due to the fact that parents with a higher level of education and greater knowledge of how the ERE could be carried out, having higher expectations, found greater deficiencies and critical issues in the design of the educational activities. The results indicate, on the other hand, according with other studies (Domina et al., 2021) that parents with a higher school level can support their children more readily both in the technical management of suitable tools, and in the supervision of the contents proposed through the ERE.

These considerations can be further explored by discussing some effects that emerge from the analysis of Model B (Figure 3) in which the effect of context variables at the level of engagement is mediated by the latent variable “impact on the family” declined in two dimensions: sharing of spaces and sharing technological tools. In general, Model B highlights that the greater the need of the family members to share spaces and tools, the less is the perceived involvement of children (β = −0.19***) in distance learning activities. In particular, where in the first model (Model A) some of the identified effects did not seem to be completely intuitive, in the second model they can be explained in greater detail. Two aspects are particularly contrived: the fact that in model A the presence of the parent working from home has no impact on engagement (β = 0.01) and also the non-impact of immigrant families (β = −0.11).

In fact, if in the model A the presence of a parent working from home has no impact on engagement, in the light of a more in-depth analysis that also includes and considers the study of the impact on the family, we note that when there is a parent working from home, the impact on the family increases in terms of sharing spaces and technological equipment (β = 0.49***), probably because family members are “forced” for reasons of work and/or support for children's learning to physically share the same spaces and tools. In the study of Di Pietro et al. (2020), for example, it has debated the role that a more well-equipped home environment, in terms of greater autonomy of spaces, has in facilitating learning of children in ERE. If the parent working from home can facilitate the support of children in ERE more closely, understanding in real time the needs that arise and proposing timely solutions (Lagomarsino et al., 2020), it is also true that the emergency situation significantly increased the risk of psycho-physical stress of parents with a potential negative effect on the well-being of children in ERE (Spinelli et al., 2020). Therefore, if the presence of the parent impacts “negatively” on the family for the reasons described, on the contrary, it positively impacts on the engagement, removing the initial effect that apparently could seem insignificant.

The non-impact of immigrant families appears at odds with the literature regarding the impact of Covid-19 on migrant families. Darmody et al. (2021), after pointing out the limited empirical evidence available on the impact of the COVID-19 pandemic on migrant/refugee/asylum-seeking children, highlight how the pandemic has widened pre-existing socio-economic inequalities (Dustmann et al., 2012; Entorf, 2015) and, in particular, educational inequalities, with dramatic consequences on vulnerable groups, such as children with special educational needs and migrant children. Similar conclusions are achieved by Bond (2020b), in her systematic review on K-12 research on teaching and learning during the COVID-19 pandemic, who points out that, even though little research has focused on migrant students, support for migrants and refugees is one of the priority topics highlighted in literature. Amongst the reasons that led to this worsening of the situation of student migrants forced to home-schooling during the pandemic, the literature highlights several factors, including the educational attainment of their parents, who are less familiar with the curriculum and with the host country language (Smyth et al., 2009; Lehmann et al., 2021); the lack of educational technology and, accordingly, reduced opportunities to engage in online education (Bayrakdar and Guveli, 2020; Popyk, 2020; Primdahl et al., 2020; Save The Children, 2020); the scarce availability of a quiet place to study (Kluge et al., 2020; Darmody et al., 2021; Lehmann et al., 2021); the socioeconomic factors that generally penalize migrants (Dustmann et al., 2012), which were found to be dramatic during the pandemic and lockdown periods (Kluge et al., 2020); the negative impact on learning engagement and academic progress of students (Mohan et al., 2020; Darmody et al., 2021; Lehmann et al., 2021).

All these studies have widely demonstrated the impact of COVID-19 pandemic on families with migrant backgrounds and, specifically to our analysis, the negative impact on students' engagement. By taking into account the number of surveys received by families with migrant backgrounds, it emerges that it is unrepresentative of the actual situation in the Italian schools. In fact, comparing data on the presence of pupils of foreign origin in Italian schools (data updated to 31/08/2019; source: Italian Ministry for Education), it emerges how the sample of respondents is extremely small: preschools: 11.4% (compared to a percentage of respondents equal to: 1.61%) primary schools: 11.5% (compared to a percentage of respondents equal to: 1.44%) lower secondary schools: 10.5% (against a response rate of: 1.07%) secondary schools: 7.4% (against a percentage of respondents equal to: 1.12%) It is therefore highly likely that the data for migrant families are in fact not statistically significant, and for this reason the models presented in this paper do not provide statistically appreciable indications of the impact that the pandemic had on these families. Although not explicitly investigated in our research, the low number of responses would seem to be a consequence of what was said earlier about the socio-economic and cultural gap between families with a migrant background and native families. Specifically, migrant families may have met problems in responding to the questionnaire due to their difficulties in accessing information technology, their educational attainment, and their difficulties with the Italian language.

Furthermore, if we look at the school level of students, we notice that as the school level increases, engagement increases (β = 0.31***) and the impact on the family decreases (β = −2.43***). Intuitively, we could hypothesize that older children impact less on the sharing of spaces and tools, as they are less dependent in terms of educational support from parents and more autonomous in the use of technology.

The data also reveal a lower suffering of families in Northern Italy respect to the impact on the family (β = −0.19**), compared to those in the Center, probably due to a better socio-cultural condition and a greater propensity to consider distance learning as a valid alternative during the lockdown period. Otherwise, for families in south Italy, no significant data emerges in comparison with the center of the Italian territory.

As for the explanatory variable “age of parents,” the model A shows a non-significant effect on perceived engagement (β = −0.03). In fact, model B highlights how the direct negative effect as the age of the parents increases (β = −0.2**) is balanced by a positive indirect effect (*ab*_*p*_*age*_*L*_*impact* = 0.2**) given by the decrease in the impact on the family (β = −1.06**).

Compared to the level of education of the parents, in the model A we observed that as the parents 'school level increases, engagement decreases (β = −0.47***). The second model, shows that the total negative effect of parents' school level on engagement is the sum of a direct effect (β = −0.17*) and of an indirect effect (*ab*_*p*_*school*_*level*_*L*_*impact* = −0.13*) on the engagement. Specifically, the indirect effect highlights the more the parents' school level increases and the more they increase the requests in terms of sharing tools and space (β = 0.7***). It is plausible that parents with a higher level of schooling, as well as professionals, have done more working from home than parents who are self-employed or have a lower level of schooling. Consequently, parents who have been working remotely from home for a long time have had the opportunity to observe their children more closely during activities in ERE, with a twofold consequence. On the one hand, being more competent due to their level of schooling, they were more sensitive in understanding the advantages and disadvantages of the didactic approach used remotely, on the other hand, remaining in the household they had more difficulties in sharing both the technological equipment and the physical spaces.

If previously the reading of this data without the impact could lead us to an interpretation that parents with a higher school level have a more negative perception of ERE, in reality, this value of the negative impact on engagement is not only due to a direct effect but it is also due to the indirect effect, that is to say the increase in the impact on the family that the parent with the highest level of education has.

## 5. Conclusion

In this paper we have examined how the student engagement construct, too often misused, poorly understood, and overgeneralized, during the period of the pandemic emergency is influenced by a variety of factors such as the family environment, the didactic approach and the student attitudes toward the remote learning activities. This with the intention of emphasizing how the construct of engagement, defined as a learner's interest and participation in an educational initiative, is directly related to favorable instructional strategies, supportive family characteristics and positive affective, cognitive-behavioral attitudes. Working collaboratively using online communication tools and building effective cooperative activities, in the specific context of the forced distance, has been found to be extremely important for student engagement. Besides, we found that the perception of interviewed parents about the student engagement depends on their effective presence and support at home, according to working from home practices, or to their school level. Moreover, the level of student's engagement reflects the parents' perception of their affective and cognitive-behavioral attitudes. So it is notable that students' engagement is related to their approach to learning processes: curiosity, interest and collaboration are important antecedents of a more involvement, such as to be restless, worried or emotionally volatile could impact differently on the level of engagement. These results suggest many interesting implications that should be addressed in the present and in the future in Italy, and in all countries involved in the pandemic, if we want to promote student engagement also during the remote learning and prevent the onset of cognitive-behavioral and affective problems linked to disengagement. Families and schools need to have correct information and guidelines about the best way to establish positive behavior support and a conducive environment that positively affects their personal and student's well being. A limitation of this study can be given by the fact that there are certain factors that have to be dealt with more in depth, with particular regard to alternative and appropriate educational suggestions to make students more engaged. At the same time, starting from the theoretical framework of Zhu et al. (2016), it could be interesting in a future study to reflect on the engagement as one of the main key factors to be improved to make ERE a multidirectional interactive learning experience based on a technology-enriched environment. Furthermore, in depth investigation of the family impact is necessary, in order to better understand the relationships between specific variables and students' engagement. Among the others, our analysis of data regarding families with migrant background has produced findings which do not fit with evidence from the several empirical studies on the subject. We argue that this is due to the limited number of questionnaires filled by migrant families, thus making data not statistically significant. Nevertheless, this reveals a dramatic fact: not only children from migrant families have been penalized by ERE more than native children, but these families have been widely excluded from surveys of the impact that ERE had on them, constituting a serious additional element of social exclusion for these families.

## Data Availability Statement

The raw data supporting the conclusions of this article will be made available by the authors, without undue reservation.

## Ethics Statement

The studies involving human participants were reviewed and approved by Commission for Ethics and Integrity in Research of the National Research Council on Italy. The participants provided their written informed consent to participate in this study.

## Author Contributions

AC: conceptualization, investigation, methodology, supervision, and writing—original draft. MA: conceptualization, investigation, project administration, and supervision. VB: conceptualization, investigation, project administration, supervision, writing—review, and editing. GC: conceptualization, investigation, and supervision. GF: conceptualization, investigation, supervision, writing—review, and editing. MG: conceptualization, data curation, formal analysis, investigation, methodology, supervision, visualization, and writing—original draft. LF: conceptualization, investigation, supervision, and writing—original draft [according to the CRediT–Contributor Roles Taxonomy (https://casrai.org/credit/)]. All authors contributed to the article and approved the submitted version.

## Conflict of Interest

The authors declare that the research was conducted in the absence of any commercial or financial relationships that could be construed as a potential conflict of interest.

## Publisher's Note

All claims expressed in this article are solely those of the authors and do not necessarily represent those of their affiliated organizations, or those of the publisher, the editors and the reviewers. Any product that may be evaluated in this article, or claim that may be made by its manufacturer, is not guaranteed or endorsed by the publisher.

## References

[B1] Affouneh S. Salha S. Khlaif Z. (2020). Designing quality e-learning environments for emergency remote teaching in coronavirus crisis. Interdiscip. J. Virtual Learn. Med. Sci. 11, 135–137. 10.30476/ijvlms.2020.86120.1033

[B2] Ato M. López-García J. J. Benavente A. (2013). Un sistema de clasificación de los diseños de investigación en psicología. Anal. Psicol. 29, 1038–1059. 10.6018/analesps.29.3.178511

[B3] Bayrakdar S. Guveli A. (2020). Inequalities in Home Learning and Schools' Provision of Distance Teaching During School Closure of COVID-19 Lockdown in the UK. Technical report, ISER Working Paper Series.

[B4] Benigno V. Caruso G. Chifari A. Ferlino L. Fulantelli G. Gentile M.. (2021). Le famiglie italiane e la didattica a distanza durante l'emergenza: una prima riflessione. Biblioteche Oggi Trends 6, 111–121.

[B5] Bergdahl N. Nouri J. Fors U. Knutsson O. (2020). Engagement, disengagement and performance when learning with technologies in upper secondary school. Comput. Educ. 149:103783. 10.1016/j.compedu.2019.103783

[B6] Bolliger D. U. Martin F. (2018). Instructor and student perceptions of online student engagement strategies. Distance Educ. 39, 568–583. 10.1080/01587919.2018.1520041

[B7] Bond M. (2020a). Facilitating student engagement through the flipped learning approach in k-12: a systematic review. Comput. Educ. 151:103819. 10.1016/j.compedu.2020.103819

[B8] Bond M. (2020b). Schools and emergency remote education during the covid-19 pandemic: a living rapid systematic review. Asian J. Distance Educ. 15, 191–247. 10.5281/zenodo.4425683

[B9] Bond M. Bedenlier S. (2019). Facilitating student engagement through educational technology: towards a conceptual framework. J. Interactive Media Educ. 2019. 11, 1–14. 10.5334/jime.528

[B10] Bozkurt A. Sharma R. C. (2020). Emergency remote teaching in a time of global crisis due to coronavirus pandemic. Asian J. Distance Educ. 15, i–vi. 10.5281/zenodo.3778083

[B11] Brom C. Lukavský J. Greger D. Hannemann T. Straková J. Švaříček R. (2020). Mandatory home education during the COVID-19 lockdown in the czech republic: a rapid survey of 1st-9th graders' parents. Front. Educ. 5:103. 10.3389/feduc.2020.00103

[B12] Darmody M. Smyth E. Russell H. (2021). Impacts of the COVID-19 control measures on widening educational inequalities. Young 29, 366–380. 10.1177/11033088211027412

[B13] Di Pietro G. Biagi F. Costa P. Karpiński Z. Mazza J. (2020). The Likely Impact of COVID-19 on Education: Reflections Based on the Existing Literature and Recent International Datasets, Vol. 30275. Luxembourg: Publications Office of the European Union.

[B14] Domina T. Renzulli L. Murray B. Garza A. N. Perez L. (2021). Remote or removed: Predicting successful engagement with online learning during COVID-19. Socius 7:237802312098820. 10.1177/2378023120988200

[B15] Dustmann C. Frattini T. Lanzara G. Algan Y. (2012). Educational achievement of second-generation immigrants: an international comparison. Econ. Policy 27, 143–185. 10.1111/j.1468-0327.2011.00275.x

[B16] Entorf H. (2015). Migrants and educational achievement gaps. IZA World Labor. April 2015, 1–10. 10.15185/izawol.146

[B17] Fugate J. M. B. Macrine S. L. Cipriano C. (2018). The role of embodied cognition for transforming learning. Int. J. Sch. Educ. Psychol. 7, 274–288. 10.1080/21683603.2018.1443856

[B18] GDPR (2016). Available online at: https://eur-lex.europa.eu/eli/reg/2016/679/oj

[B19] Gentile M. Benigno V. Caruso G. Chifari A. Ferlino L. Fulantelli G.. (2021). Italian parents' perception about learning practices and educational effectiveness of remote schooling during the first lockdown. Qwerty-Open and Interdisciplinary. J. Technol. Cult. Educ. 16.

[B20] Gigli A. (2020). Essere genitori ai tempi del covid-19: disagi, bisogni, risorse. i primi dati di una rilevazione. Infanzia, famiglie, servizi educativi e scolastici nel COVID-19, 18.

[B21] Hapsari S. M. Sugito S. Fauziah P. Y. (2020). Parent's involvement in early childhood education during the covid-19 pandemic period. J. Pendidikan Progresif 10, 298–311. 10.23960/jpp.v10.i2.202014

[B22] Hoerger M. Currell C. (2012). Ethical issues in internet research, in APA Handbook of Ethics in Psychology, Vol. 2: Practice, Teaching, and Research (Washington, DC: American Psychological Association), 385–400. 10.1037/13272-018

[B23] Hsiao Y.-C. (2021). Impacts of course type and student gender on distance learning performance: a case study in taiwan. Educ. Inf. Technol. 1–16. 10.1007/s10639-021-10538-8 33867809 PMC8043437

[B24] Khlaif Z. N. Salha S. Kouraichi B. (2021). Emergency remote learning during COVID-19 crisis: students' engagement. Edu. Inf. Technolo. 1–23. 10.1007/s10639-021-10566-4 33935578 PMC8077864

[B25] Kish L. (1987). Statistical Design for Research. Hoboken, NJ: John Wiley & Sons, Inc. 10.1002/0471725196

[B26] Kline R. B. (2015). Principles and Practice of Structural Equation Modeling. New York, NY: Guilford Publications.

[B27] Kluge H. H. P. Jakab Z. Bartovic J. D'Anna V. Severoni S. (2020). Refugee and migrant health in the COVID-19 response. Lancet 395, 1237–1239. 10.1016/S0140-6736(20)30791-1 32243777 PMC7270965

[B28] Korlat S. Kollmayer M. Holzer J. Lüftenegger M. Pelikan E. R. Schober B.. (2021). Gender differences in digital learning during COVID-19: competence beliefs, intrinsic value, learning engagement, and perceived teacher support. Front. Psychol. 12:637776. 10.3389/fpsyg.2021.637776 33868109 PMC8043960

[B29] Lagomarsino F. Coppola I. Parisi R. Rania N. (2020). Care tasks and new routines for italian families during the covid-19 pandemic: perspectives from women. Italian Sociol. Rev. 10, 2020. 10.13136/isr.v10i3S.401

[B30] Lehmann S. Skogen J. C. Haug E. Mæland S. Fadnes L. T. Sandal G. M.. (2021). Perceived consequences and worries among youth in norway during the COVID-19 pandemic lockdown. Scand. J. Public Health 49, 755–765. 10.1177/1403494821993714 33645323 PMC8521367

[B31] Martin F. Bolliger D. U. (2018). Engagement matters: student perceptions on the importance of engagement strategies in the online learning environment. Online Learn. 22, 205–222. 10.24059/olj.v22i1.1092

[B32] Mohan G. McCoy S. Carroll E. Mihut G. Lyons S. Domhnaill C. M. (2020). Learning for all? second-level education in ireland during COVID-19. Technical report.

[B33] Ndhine F. (2020). Roles of Parents in Their Child's Education During COVID-19 and Thereafter. Nova Pioneer. Available online at: https://www.novapioneer.com/kenya/blog/roles-of-parents-in-their-childs-education-during-covid-19-and-thereafter/

[B34] Newmann F. M. Wehlage G. Lamborn S. D. (1992). The significance and sources of student engagement, in Student Engagement and Achievement in American Secondary Schools, Chapter 1, ed Newmann F. M. (New York, NY: Teachers College Press), 11–39.

[B35] Parmigiani D. Benigno V. Giusto M. Silvaggio C. Sperandio S. (2020). E-inclusion: online special education in italy during the COVID-19 pandemic. Technol. Pedagogy Educ. 30, 111–124. 10.1080/1475939X.2020.1856714

[B36] Pellegrini M. Maltinti C. (2020). “School never stops”: Measures and experience in italian schools during the COVID-19 lockdown. Best Evid. Chin. Edu. 5, 649–663. 10.15354/bece.20.or021

[B37] Popyk A. (2020). The impact of distance learning on the social practices of schoolchildren during the COVID-19 pandemic: reconstructing values of migrant children in poland. Eur. Soc. 23(Sup1.):S530–S544. 10.1080/14616696.2020.1831038

[B38] Primdahl N. L. Borsch A. S. Verelst A. Jervelund S. S. Derluyn I. Skovdal M. (2020). ‘it's difficult to help when i am not sitting next to them’: How COVID-19 school closures interrupted teachers' care for newly arrived migrant and refugee learners in denmark. Vulnerable Children Youth Stud. 16, 75–85. 10.1080/17450128.2020.1829228

[B39] R Core Team (2018). R: A Language and Environment for Statistical Computing. Vienna: R Foundation for Statistical Computing.

[B40] Rosseel Y. (2012). lavaan: An R package for structural equation modeling. J. Stat. Softw. 48, 1–36. 10.18637/jss.v048.i0225601849

[B41] Save The Children (2020). COVID-19: Operational Guidance for Migrant Displaced Children. Technical report.

[B42] Schermelleh-Engel K. Moosbrugger H. Müller H. (2003). Evaluating the fit of structural equation models: tests of significance and descriptive goodness-of-fit measures. Methods Psychol. Res. Online 8, 23–74.

[B43] Smyth E. Darmody M. McGinnity F. Byrne D. (2009). Adapting to diversity: Irish schools and newcomer students,” in ESRI. Dublin.

[B44] Spinelli M. Lionetti F. Pastore M. Fasolo M. (2020). Parents' stress and children's psychological problems in families facing the COVID-19 outbreak in italy. Front. Psychol. 11:1713. 10.3389/fpsyg.2020.01713 32719646 PMC7350926

[B45] United Nations Educational (2021). Supporting Learning Recovery One Year Into COVID-19: The Global Education Coalition in Action. Technical report.

[B46] Wong A. Chong S. (2018). Modelling adult learners' online engagement behaviour: proxy measures and its application. J. Comput. Educ. 5, 463–479. 10.1007/s40692-018-0123-z

[B47] Yu Z. (2021). The effects of gender, educational level, and personality on online learning outcomes during the COVID-19 pandemic. Int. J. Educ. Technol. Higher Educ. 18:14. 10.1186/s41239-021-00252-3 PMC8016506 34778520

[B48] Zhu Z.-T. Yu M.-H. Riezebos P. (2016). A research framework of smart education. Smart Learn. Environ. 3:4. 10.1186/s40561-016-0026-2

