# Supplementary material for: Distance Learning During the First Lockdown: Impact on the Family and Its Effect on Students' Engagement
Source: Front Psychol. 2021 Nov 11;12:762213. doi: 10.3389/fpsyg.2021.762213 (PMC8632369; doi:10.3389/fpsyg.2021.762213)
Supplement: Supplementary file 1 [file Data_Sheet_1.PDF]

La famiglia e la scuola ai tempi del Covid-19

This file contains the questionnaire “La famiglia e la scuola ai tempi del Covid-19” (Family and school at the time of the of Covid-19) both in its original Italian form and in its English translation.

The research team composed of: Vincenza Benigno, Giovanni Caruso, Antonella Chifari, Lucia Ferlino, Giovanni Fulantelli, Manuel Gentile and Mario Allegra from the Institute for Educational Technologies - National Research Council of Italy, wants to give voice to families to understand how Emergency Remote Education (ERE) was managed. In particulare, the survbey is aimed to investigate how the technological resources present in the home have been managed, whether they have been sufficient, whether family organization and habits have changed, what kind of relations have been established with schools and what difficulties have emerged.
The questionnaire consists of three sections.

1 Sezione Anagrafica

1.1 Dati del genitore che compila il questionario

1.1.1 Genere

Scegli **solo una** delle seguenti:

- Femmina
- Maschio

1.1.2 Età anagrafica *

Scegliere solo una delle seguenti voci
Scegli **solo una** delle seguenti:

- <20
- 20-24
- 25-29
- 30-34
- 35-39
- 40-44
- 45-49
- 50-54
- >54

1.1.3 Nazionalità *

Scegliere solo una delle seguenti voci
Scegli **solo una** delle seguenti:

1.1.4 Cittadinanza *

Scegliere solo una delle seguenti voci
Scegli **solo una** delle seguenti:

- Italiana
- Immigrato di prima generazione
- Immigrato di seconda generazione
- Altro

1.1.5 Livello scolare*

Scegliere solo una delle seguenti voci
Scegli **solo una** delle seguenti:

- Nessuno
- Licenza elementare
- Licenza media

- Diploma di istruzione secondaria superiore
- Laurea di primo livello
- Laurea specialistica o a ciclo unico

1.1.6 Stato occupazionale *

Scegliere solo una delle seguenti voci
Scegli **solo una** delle seguenti:

- Occupato
- Disoccupato
- Pensionato
- Altro

1.1.7 Sta svolgendo la sua attività lavorativa in modalità smart working? *

Rispondere solo se le seguenti condizioni sono rispettate:
La risposta era 'Occupato' Alla domanda '8 [AN05]' (Stato occupazionale)
Scegli **solo una** delle seguenti:

- Sì
- No

1.2 Dati dell'altro genitore

1.2.1 Età anagrafica

Scegliere solo una delle seguenti voci
Scegli **solo una** delle seguenti:

- <20
- 20-24
- 25-29
- 30-34
- 35-39
- 40-44
- 45-49
- 50-54
- >54

1.2.2 Nazionalità

Scegliere solo una delle seguenti voci
Scegli **solo una** delle seguenti:

1.2.3 Cittadinanza

Scegliere solo una delle seguenti voci
Scegli **solo una** delle seguenti:

- Italiana
- Immigrato di prima generazione
- Immigrato di seconda generazione
- Altro

1.2.4 Livello scolare

Scegliere solo una delle seguenti voci
Scegli **solo una** delle seguenti:

- Nessuno
- Licenza elementare
- Licenza media
- Diploma di istruzione secondaria superiore
- Laurea di primo livello
- Laurea specialistica o a ciclo unico

1.2.5 Stato occupazionale

Scegliere solo una delle seguenti voci
Scegli **solo una** delle seguenti:

- Occupato
- Disoccupato
- Pensionato
- Altro

1.2.6 Sta svolgendo la sua attività lavorativa in modalità smart working? *

Rispondere solo se le seguenti condizioni sono rispettate:
La risposta era 'Occupato' Alla domanda '15 [AN09]' (Stato occupazionale)
Scegli **solo una** delle seguenti:

- Sì
- No

1.3 Dati del nucleo familiare

1.3.1 Provincia di residenza *

Scegliere solo una delle seguenti voci
Scegli **solo una** delle seguenti:

1.3.2 Per la gestione dei figli è stato necessario ricorrere ad un sostegno esterno? *

Scegli **solo una** delle seguenti:

- Sì
- No

1.3.3 Che tipo di supporto? *

Rispondere solo se le seguenti condizioni sono rispettate:
La risposta era 'Sì' Alla domanda '19 [AN11]' (Per la gestione dei figli è stato necessario ricorrere ad un sostegno esterno?)
Scegliere una o più delle seguenti opzioni
Scegliere **tutte** le corrispondenti:

- Nonni
- Baby sitter
- Parenti
- Amici
- Altro:

1.3.4 Composizione nucleo familiare *

	**Numero**
**Adulti**	
**Figli**	
**di cui in età scolare (dalla scuola dell'infanzia alla scuola secondaria di secondo grado)**	

ATTENZIONE!

Qualora avesse più di un figlio in età scolare (dalla scuola dell'infanzia alla scuola secondaria di secondo grado) per cui è prevista la compilazione di più schede, una per figlio, risponda a ciascuna domanda facendo riferimento alla specifica situazione vissuta da ciascuno di essi indipendentemente l'uno dall'altro.

1.4 Sezione Tecnologica

1.4.1 A casa, disponete di strumenti tecnologici (pc, tablet)? *

Scegli **solo una** delle seguenti:

- Sì
- No

1.4.2 Di che tipo di strumenti tecnologici siete in possesso? *

Rispondere solo se le seguenti condizioni sono rispettate:
La risposta era 'Sì' Alla domanda '23 [TEC01]' (A casa, disponete di strumenti tecnologici (pc, tablet)?)
Scegliere una o più delle seguenti opzioni
Scegliere **tutte** le corrispondenti:

- PC portatile
- Tablet
- PC fisso
- Stampante
- Altro:

1.4.3 Numero PC portatili posseduti ed utilizzati abitualmente *

Rispondere solo se le seguenti condizioni sono rispettate:
La risposta era Alla domanda '24 [TEC01D]' (Di che tipo di strumenti tecnologici siete in possesso?)
Scegliere solo una delle seguenti voci
Scegli **solo una** delle seguenti:

- 1
- 2
- 3
- 4
- 5
- 6 o più

1.4.4 Numero Tablet posseduti ed utilizzati abitualmente *

Rispondere solo se le seguenti condizioni sono rispettate:
La risposta era Alla domanda '24 [TEC01D]' (Di che tipo di strumenti tecnologici siete in possesso?)
Scegliere solo una delle seguenti voci
Scegli **solo una** delle seguenti:

- 1
- 2
- 3
- 4
- 5
- 6 o più

1.4.5 Numero PC fissi posseduti ed utilizzati abitualmente *

Rispondere solo se le seguenti condizioni sono rispettate:
La risposta era Alla domanda '24 [TEC01D]' (Di che tipo di strumenti tecnologici siete in possesso?)
Scegliere solo una delle seguenti voci
Scegli **solo una** delle seguenti:

- 1
- 2
- 3
- 4
- 5

- 6 o più

1.4.6 Suo/a figlio/a ha potuto disporre sin da subito di tutta la dotazione tecnologica necessaria per inserirsi tempestivamente nei corsi a distanza?*

Scegli **solo una** delle seguenti:

- Sì
- No

1.4.7 La scuola ha provveduto *

Rispondere solo se le seguenti condizioni sono rispettate:
La risposta era 'No' Alla domanda '28 [TEC02]' (Suo/a figlio/a ha potuto disporre sin da subito di tutta la dotazione tecnologica necessaria per inserirsi tempestivamente nei corsi a distanza?)
Scegliere solo una delle seguenti voci
Scegli **solo una** delle seguenti:

- In nessun modo
- Fornendo alla famiglia un tablet come da indicazioni ministeriali
- Fornendo alla famiglia ulteriori dispositivi tecnologici necessari
- Fornendo alla famiglia l'accesso gratuito alla rete della scuola per la connessione Internet
- Fornendo alla famiglia l'accesso alla rete internet attraverso l'acquisto di abbonamenti alla rete cellulare

1.4.8 Rispetto alla strumentazione presente o meno in casa prima dell'emergenza, ha dovuto attrezzarsi con altra dotazione tecnologica? *

Scegli **solo una** delle seguenti:

- Sì
- No

1.4.9 Indicare il numero delle strumentazioni acquisite *

Rispondere solo se le seguenti condizioni sono rispettate:
La risposta era 'Sì' Alla domanda '30 [TEC03]' (Rispetto alla strumentazione presente o meno in casa prima dell'emergenza, ha dovuto attrezzarsi con altra dotazione tecnologica?)

	**Numero**
**PC portatile**	
**Tablet**	
**PC fisso**	
**Stampante**	
**Altro**	

1.4.10 Che tipo di connessione avete in casa? *

Scegliere solo una delle seguenti voci
Scegli **solo una** delle seguenti:

- adsl
- fibra ottica
- connessione tramite rete cellulare (con router o smartphone)

1.4.11 È stato necessario cambiare il tipo di connessione / l'offerta commerciale a seguito dell'emergenza? *

Scegli **solo una** delle seguenti:

- Sì
- No

1.4.12 Indichi il suo livello di competenza relativo *

Scegliere la risposta appropriata per ciascun elemento:

	nullo	basso	medio	buono	elevato
**all'uso dei più diffusi strumenti tecnologici (es. computer, tablet)**					
**all'uso di Internet e dei suoi applicativi (e-mail, web)**					
**agli ambienti utilizzati da suo/a figlio/a nella DaD (registro elettronico, piattaforme didattiche, sistemi per la videoconferenza e per la comunicazione)**					

2 Scheda Figlio

2.1.1 Genere

Scegli **solo una** delle seguenti:

- Femmina
- Maschio

2.1.2 Livello scolare *

Scegliere solo una delle seguenti voci
Scegli **solo una** delle seguenti:

- Infanzia
- Primaria (primo ciclo 1-2)
- Primaria (secondo ciclo 3-5)
- Scuola secondaria di I grado
- Scuola secondaria di II grado

2.1.3 Per favore, indicate se vostro/a figlio/a presenta:*

Scegliere la risposta appropriata per ciascun elemento:

	Si	No
**Disabilità**		
**DSA**		
**Altri BES**		

2.1.4 Che tipo di scuola frequenta suo/a figlio/a? *

Rispondere solo se le seguenti condizioni sono rispettate:
La risposta era 'Scuola secondaria di II grado' Alla domanda '36 [F0101]' (Livello scolare)
Scegliere solo una delle seguenti voci
Scegli **solo una** delle seguenti:

- Liceo
- Istituto Tecnico
- Istituto Professionale

2.1.5 Prima dell'emergenza Covid-19, i docenti usavano le tecnologie per la didattica (es. LIM, computer, tablet, robotica, sistemi per la messaggistica) ? *

Rispondere solo se le seguenti condizioni sono rispettate:
La risposta era NO 'Si' Alla domanda '37 [F0102]' ((Disabilità))
Scegli **solo una** delle seguenti:

- Sì

- No

2.1.6 Indica quali tecnologie utilizzavano i docenti *

Rispondere solo se le seguenti condizioni sono rispettate:
La risposta era 'Sì' Alla domanda '39 [F0104]' (Prima dell'emergenza Covid-19, i docenti usavano le tecnologie per la didattica (es. LIM, computer, tablet, robotica, sistemi per la messaggistica) ?)
Scegliere una o più delle seguenti opzioni
Scegliere **tutte** le corrispondenti:

- LIM
- computer
- tablet
- robotica educativa
- registro elettronico
- sistemi per la messaggistica/comunicazione
- sistemi per la didattica a distanza

2.1.7 Prima dell'emergenza Covid-19, i docenti hanno proposto attività didattiche a distanza? *

Scegli **solo una** delle seguenti:

- Sì
- No

2.1.8 Durante l'emergenza Covid-19, i docenti hanno proposto delle attività a distanza? *

Scegli **solo una** delle seguenti:

- Sì
- No

2.1.9 Che tipo di disabilità (ai sensi della Legge 104/92) presenta suo/a figlio/a? *

Rispondere solo se le seguenti condizioni sono rispettate:
La risposta era 'Sì' Alla domanda '42 [F0105]' (Durante l'emergenza Covid-19, i docenti hanno proposto delle attività a distanza?) *e* La risposta era 'Si' Alla domanda '37 [F0102]' ((Disabilità))
Scegliere solo una delle seguenti voci
Scegli **solo una** delle seguenti:

- Intellettiva
- Motoria
- Sensoriale (visiva)
- Sensoriale (uditiva)
- Spettro dell'autismo
- Pluridisabilità

2.1.10 Prima dell’emergenza Covid-19 suo/a figlio/a da chi era seguito e per quante ore settimanali? *

Rispondere solo se le seguenti condizioni sono rispettate:
La risposta era 'Sì' Alla domanda '42 [F0105]' (Durante l’emergenza Covid-19, i docenti hanno proposto delle attività a distanza?) *e* La risposta era 'Si' Alla domanda '37 [F0102]' ((Disabilità))
Scegliere una o più delle seguenti opzioni
Scegliere **tutte** le corrispondenti:

- Dal docente di sostegno
- Da un OSE (operatore socio-educativo)
- Dai docenti della classe

2.1.11 Ore docente di sostegno *

Rispondere solo se le seguenti condizioni sono rispettate:
La risposta era Alla domanda '44 [DIS0103]' (Prima dell’emergenza Covid-19 suo/a figlio/a da chi era seguito e per quante ore settimanali?)
Scegliere solo una delle seguenti voci
Scegli **solo una** delle seguenti:

- 1
- ..
- 36

2.1.12 Ore OSE *

Rispondere solo se le seguenti condizioni sono rispettate:
La risposta era Alla domanda '44 [DIS0103]' (Prima dell’emergenza Covid-19 suo/a figlio/a da chi era seguito e per quante ore settimanali?)
Scegliere solo una delle seguenti voci
Scegli **solo una** delle seguenti:

- 1
- …
- 36

2.1.13 Ore docenti della classe *

Rispondere solo se le seguenti condizioni sono rispettate:
La risposta era Alla domanda '44 [DIS0103]' (Prima dell’emergenza Covid-19 suo/a figlio/a da chi era seguito e per quante ore settimanali?)
Scegliere solo una delle seguenti voci
Scegli **solo una** delle seguenti:

- 1
- …
- 36

2.1.14 Prima dell'emergenza Covid-19 suo/a figlio/a seguiva *

Rispondere solo se le seguenti condizioni sono rispettate:
La risposta era 'Sì' Alla domanda '42 [F0105]' (Durante l'emergenza Covid-19, i docenti hanno proposto delle attività a distanza?) *e* La risposta era 'Si' Alla domanda '37 [F0102]' ((Disabilità))
Scegliere la risposta appropriata per ciascun elemento:

	**mai**	**una volta al mese**	**una volta a settimana**	**due/tre volte a settimana**	**tutti i giorni**
**Un percorso individualizzato dentro la classe ma con scarsa interazione con i compagni di classe**					
**Un percorso individualizzato fuori dalla classe**					
**Un percorso individualizzato dentro la classe con frequenti momenti di inclusione**					

2.1.15 Suo/a figlio/a necessita di ausili didattici (es. per scrivere, leggere, comunicare)? *

Rispondere solo se le seguenti condizioni sono rispettate:
La risposta era 'Sì' Alla domanda '42 [F0105]' (Durante l'emergenza Covid-19, i docenti hanno proposto delle attività a distanza?) *e* La risposta era 'Si' Alla domanda '37 [F0102]' ((Disabilità))
Scegli **solo una** delle seguenti:

- Sì
- No

2.1.16 Indichi quali *

Rispondere solo se le seguenti condizioni sono rispettate:
La risposta era 'Sì' Alla domanda '49 [DIS0105]' (Suo/a figlio/a necessita di ausili didattici (es. per scrivere, leggere, comunicare)?)
Scegliere una o più delle seguenti opzioni
Scegliere **tutte** le corrispondenti:

- Barra braille
- Riconoscimento vocale
- Sintesi vocale
- Video-ingranditori
- Sistemi tecnologici specifici per non udenti/ipoacusici
- Comunicatori
- Computer
- Altro:

2.1.17 Dalla chiusura della scuola, quanto tempo è passato per l'avvio della DaD? *

Rispondere solo se le seguenti condizioni sono rispettate:
La risposta era 'Sì' Alla domanda '42 [F0105]' (Durante l’emergenza Covid-19, i docenti hanno proposto delle attività a distanza?)
Scegliere solo una delle seguenti voci
Scegli **solo una** delle seguenti:

- Una settimana
- Due settimane
- Tre settimane
- Altro

2.1.18 I docenti hanno avviato le lezioni in modalità DaD? *

Rispondere solo se le seguenti condizioni sono rispettate:
La risposta era 'Sì' Alla domanda '42 [F0105]' (Durante l’emergenza Covid-19, i docenti hanno proposto delle attività a distanza?)
Scegliere solo una delle seguenti voci
Scegli **solo una** delle seguenti:

- Sì, tutti
- Si, la maggior parte
- Sì, alcuni

2.1.19 Siete stati contattati dalla scuola e informati sulle modalità della DaD? *

Rispondere solo se le seguenti condizioni sono rispettate:
La risposta era 'Sì' Alla domanda '42 [F0105]' (Durante l’emergenza Covid-19, i docenti hanno proposto delle attività a distanza?)
Scegli **solo una** delle seguenti:

- Sì
- No

2.1.20 In che modo siete stati contattati? *

Rispondere solo se le seguenti condizioni sono rispettate:
La risposta era 'Sì' Alla domanda '53 [F0108]' (Siete stati contattati dalla scuola e informati sulle modalità della DaD?)
Scegliere una o più delle seguenti opzioni
Scegliere **tutte** le corrispondenti:

- Telefonata/videochiamata
- Invio lettera tramite posta elettronica da parte del dirigente scolastico
- SMS
- Messaggio su WhatsApp o altro sistema di messaggistica
- Altro:

2.1.21 La scuola vi ha comunicato cambiamenti nella programmazione dovuti alla nuova modalità di DaD? *

Rispondere solo se le seguenti condizioni sono rispettate:
La risposta era 'Sì' Alla domanda '42 [F0105]' (Durante l'emergenza Covid-19, i docenti hanno proposto delle attività a distanza?)
Scegliere solo una delle seguenti voci
Scegli **solo una** delle seguenti:

- No
- Si, solo all'inizio del periodo
- Si, periodicamente

2.1.22 La scuola vi ha chiesto quali strumenti tecnologici per connettervi a Internet avevate a disposizione? *

Rispondere solo se le seguenti condizioni sono rispettate:
La risposta era 'Sì' Alla domanda '42 [F0105]' (Durante l'emergenza Covid-19, i docenti hanno proposto delle attività a distanza?)
Scegli **solo una** delle seguenti:

- Sì
- No

2.1.23 I docenti propongono attività in tempo reale (es. attraverso videoconferenza)? *

Rispondere solo se le seguenti condizioni sono rispettate:
La risposta era 'Sì' Alla domanda '42 [F0105]' (Durante l'emergenza Covid-19, i docenti hanno proposto delle attività a distanza?) *e* La risposta era 'Infanzia ' Alla domanda '36 [F0101]' (Livello scolare) *e* La risposta era NO 'Si' Alla domanda '37 [F0102]' ((Disabilità))
Scegli **solo una** delle seguenti:

- Sì
- No

2.1.24 Quale tipo di attività propongono? *

Rispondere solo se le seguenti condizioni sono rispettate:
La risposta era 'Sì' Alla domanda '57 [F0111]' (I docenti propongono attività in tempo reale (es. attraverso videoconferenza)?)
Scegliere una o più delle seguenti opzioni
Scegliere **tutte** le corrispondenti:

- Disegno
- Canzoni/filastrocche/poesie
- Creazione di manufatti
- Racconto di storie

- Attività motoria
- Altro:

2.1.25 È richiesta la presenza di un adulto? *

Rispondere solo se le seguenti condizioni sono rispettate:
La risposta era 'Sì' Alla domanda '57 [F0111]' (I docenti propongono attività in tempo reale (es. attraverso videoconferenza)?)
Scegliere solo una delle seguenti voci
Scegli **solo una** delle seguenti:

- Si, per avviare l'attività
- Si, per tutta la durata dell'attività
- No

2.1.26 Con la nuova modalità di DaD, l'orario settimanale delle attività didattiche è stato rimodulato? *

Rispondere solo se le seguenti condizioni sono rispettate:
La risposta era 'Sì' Alla domanda '42 [F0105]' (Durante l'emergenza Covid-19, i docenti hanno proposto delle attività a distanza?) *e* La risposta era NO 'Infanzia ' Alla domanda '36 [F0101]' (Livello scolare)
Scegli **solo una** delle seguenti:

- Sì
- No

2.1.27 Con la nuova modalità di DaD, le attività si svolgono: *

Rispondere solo se le seguenti condizioni sono rispettate:
La risposta era 'Sì' Alla domanda '60 [F0112]' (Con la nuova modalità di DaD, l'orario settimanale delle attività didattiche è stato rimodulato?)
Scegliere solo una delle seguenti voci
Scegli **solo una** delle seguenti:

- Tutti i giorni
- Quasi tutti i giorni
- Due/tre volte a settimana
- Una volta a settimana

2.1.28 Con quali strumenti e modalità i docenti stanno gestendo la DaD? *

Rispondere solo se le seguenti condizioni sono rispettate:
La risposta era 'Sì' Alla domanda '42 [F0105]' (Durante l'emergenza Covid-19, i docenti hanno proposto delle attività a distanza?)
Scegliere una o più delle seguenti opzioni
Scegliere **tutte** le corrispondenti:

- Registro elettronico

- Sistemi di videoconferenza (es. Skype, Zoom, Google Meet, Jitsi, BigBlueButton….)
- Messaggistica istantanea (es. WhatsApp, Telegram)
- Piattaforme (Gsuite for Education, Office 365, Moodle, WeSchool, etc.)
- Cartelle condivise
- App case editrici
- Altro:

2.1.29 I docenti delle diverse discipline usano lo stesso sistema di videocomunicazione per entrare in contatto e svolgere le attività didattiche con gli studenti? *

Rispondere solo se le seguenti condizioni sono rispettate:
La risposta era 'Sì' Alla domanda '42 [F0105]' (Durante l'emergenza Covid-19, i docenti hanno proposto delle attività a distanza?) *e* La risposta era NO 'Infanzia ' Alla domanda '36 [F0101]' (Livello scolare) *e* La risposta era NO 'Si' Alla domanda '37 [F0102]' ((Disabilità))
Scegli **solo una** delle seguenti:

- Sì
- No

2.1.30 Per svolgere le attività didattiche i docenti utilizzano *

Rispondere solo se le seguenti condizioni sono rispettate:
La risposta era 'Sì' Alla domanda '42 [F0105]' (Durante l'emergenza Covid-19, i docenti hanno proposto delle attività a distanza?) *e* La risposta era NO 'Infanzia ' Alla domanda '36 [F0101]' (Livello scolare)
Scegliere una o più delle seguenti opzioni
Scegliere **tutte** le corrispondenti:

- videoconferenza
- materiali audio/video (es. filmati riguardanti spiegazioni degli argomenti, lettura di racconti, cartoni animati, ecc.)
- questionari digitali (moduli da compilare direttamente online)
- questionari in formato "cartaceo" (es. foto o scansioni di documenti)
- lavagna condivisa (es. il docente agisce sul proprio dispositivo per disegnare o svolgere delle attività come se fosse alla lavagna o su un foglio e gli studenti possono intervenire in modo interattivo)
- condivisione dello schermo (es. il docente condivide lo schermo ma gli studenti possono solo visualizzare e non intervenire in modo interattivo)
- Altro:

2.1.31 In questa fase, sono state previste attività di apprendimento collaborativo (attività in gruppo, progetti, ricerche, etc.)? *

Rispondere solo se le seguenti condizioni sono rispettate:
La risposta era 'Sì' Alla domanda '42 [F0105]' (Durante l'emergenza Covid-19, i docenti hanno proposto delle attività a distanza?) *e* La risposta era NO 'Infanzia ' Alla domanda '36 [F0101]' (Livello scolare)
Scegli **solo una** delle seguenti:

- Sì
- No

2.1.32 I docenti richiedono ai bambini l’invio della realizzazione di compiti (ad es. disegni)? *

Rispondere solo se le seguenti condizioni sono rispettate:
La risposta era 'Sì' Alla domanda '42 [F0105]' (Durante l’emergenza Covid-19, i docenti hanno proposto delle attività a distanza?) *e* La risposta era 'Infanzia ' Alla domanda '36 [F0101]' (Livello scolare) *e* La risposta era NO 'Si' Alla domanda '37 [F0102]' ((Disabilità))
Scegli **solo una** delle seguenti:

- Sì
- No

2.1.33 La scuola frequentata da suo/a figlio/a prevede attività laboratoriali in orario scolastico ed extrascolastico? *

Rispondere solo se le seguenti condizioni sono rispettate:
La risposta era 'Sì' Alla domanda '42 [F0105]' (Durante l’emergenza Covid-19, i docenti hanno proposto delle attività a distanza?) *e* La risposta era 'Scuola secondaria di II grado' Alla domanda '36 [F0101]' (Livello scolare) *e* La risposta era NO 'Si' Alla domanda '37 [F0102]' ((Disabilità))
Scegli **solo una** delle seguenti:

- Sì
- No

2.1.34 Le attività sono state... *

Rispondere solo se le seguenti condizioni sono rispettate:
La risposta era 'Sì' Alla domanda '67 [F0118]' (La scuola frequentata da suo/a figlio/a prevede attività laboratoriali in orario scolastico ed extrascolastico?)
Scegliere solo una delle seguenti voci
Scegli **solo una** delle seguenti:

- sospese
- sospese, perché non è possibile svolgerle a distanza
- modificate, adattandole alla modalità a distanza
- mantenute, perché prevedevano già lo svolgimento a distanza
- Altro

2.1.35 Durante le attività didattiche in modalità DaD suo/a figlio/a chiede il suo supporto? *

Rispondere solo se le seguenti condizioni sono rispettate:
La risposta era 'Sì' Alla domanda '42 [F0105]' (Durante l’emergenza Covid-19, i docenti hanno proposto delle attività a distanza?) *e* La risposta era NO 'Infanzia ' Alla domanda '36 [F0101]' (Livello scolare)

Scegliere solo una delle seguenti voci
Scegli **solo una** delle seguenti:

- No, è autonomo
- Si, lo richiede
- Si, ma non ho le competenze necessarie per aiutarlo

2.1.36 Durante le attività didattiche in modalità DaD, suo/a figlio/a chiede supporto

Rispondere solo se le seguenti condizioni sono rispettate:
La risposta era 'Si, lo richiede' *o* 'Si, ma non ho le competenze necessarie per aiutarlo' Alla domanda '69 [F0120]' (Durante le attività didattiche in modalità DaD suo/a figlio/a chiede il suo supporto?)
Scegliere una o più delle seguenti opzioni
Scegliere **tutte** le corrispondenti:

- per avviare la lezione/attività
- per tutta la durata della lezione/attività
- per la gestione della strumentazione
- perché ha difficoltà nella comprensione dei contenuti
- perché ha difficoltà a seguire nella modalità a distanza

2.1.37 Le lezioni in modalità DaD prevedono *

Rispondere solo se le seguenti condizioni sono rispettate:
La risposta era 'Sì' Alla domanda '42 [F0105]' (Durante l'emergenza Covid-19, i docenti hanno proposto delle attività a distanza?) *e* La risposta era 'Si' Alla domanda '37 [F0102]' ((Disabilità))
Scegliere la risposta appropriata per ciascun elemento:

	**mai**	**una volta a settimana**	**due/tre volte a settimana**	**tutti i giorni**
**Un percorso individualizzato senza contatto con la classe**				
**Momenti di interazione con la classe su attività didattiche**				
**Momenti di interazione con la classe per la socializzazione**				

2.1.38 Le lezioni in modalità DaD *

Rispondere solo se le seguenti condizioni sono rispettate:
La risposta era 'Sì' Alla domanda '42 [F0105]' (Durante l'emergenza Covid-19, i docenti hanno proposto delle attività a distanza?) *e* La risposta era 'Si' Alla domanda '37 [F0102]' ((Disabilità))
Scegliere solo una delle seguenti voci
Scegli **solo una** delle seguenti:

- hanno favorito i rapporti tra suo/a figlio/a e la classe
- hanno peggiorato i rapporti tra suo/a figlio/a e la classe
- non saprei

2.1.39 Secondo lei, le lezioni in modalità DaD hanno avuto conseguenze in termini di *

Rispondere solo se le seguenti condizioni sono rispettate:
La risposta era 'Sì' Alla domanda '42 [F0105]' (Durante l'emergenza Covid-19, i docenti hanno proposto delle attività a distanza?) *e* La risposta era 'Si' Alla domanda '37 [F0102]' ((Disabilità))
Scegliere la risposta appropriata per ciascun elemento:

	molto negative - 0	1	2	3	4	5	6	7	8	9	molto positive - 10
**Apprendimento**											
**Autonomia**											
**Comunicazione**											

2.1.40 Secondo lei la DaD:

Rispondere solo se le seguenti condizioni sono rispettate:
La risposta era 'Sì' Alla domanda '42 [F0105]' (Durante l'emergenza Covid-19, i docenti hanno proposto delle attività a distanza?) *e* La risposta era NO 'Infanzia ' Alla domanda '36 [F0101]' (Livello scolare) *e* La risposta era NO 'Si' Alla domanda '37 [F0102]' ((Disabilità))
Scegliere una o più delle seguenti opzioni
Scegliere **tutte** le corrispondenti:

- sta favorendo l'acquisizione di nuovi contenuti disciplinari
- sta favorendo la conoscenza di metodi di apprendimento più accattivanti
- sta favorendo l'acquisizione di competenze digitali e tecnologiche
- è una valida opportunità di crescita per mio/a figlio/a, anche in previsione di nuovi scenari futuri

2.1.41 Esprima una valutazione rispetto all'efficacia/inefficacia *

Rispondere solo se le seguenti condizioni sono rispettate:
La risposta era 'Sì' Alla domanda '42 [F0105]' (Durante l'emergenza Covid-19, i docenti hanno proposto delle attività a distanza?)
Scegliere la risposta appropriata per ciascun elemento:

	Poco efficace - 0	1	2	3	4	5	6	7	8	9	Molto Efficace - 10
**delle attività didattiche proposte con la DaD**											
**dell'interazione e dello scambio comunicativo tra scuola-famiglia**											
**dell'interazione con gli altri genitori/rappresentanti di classe**											

Questo nuovo modo di fare scuola (DaD) di suo/a figlio/a quanto ha influito sull’ordinaria organizzazione familiare?

2.1.42 *

Rispondere solo se le seguenti condizioni sono rispettate:
La risposta era 'Sì' Alla domanda '42 [F0105]' (Durante l’emergenza Covid-19, i docenti hanno proposto delle attività a distanza?)
Scegliere la risposta appropriata per ciascun elemento:

	Per nulla - 0	1	2	3	4	5	6	7	8	9	Moltissimo - 10
nella condivisione degli spazi domestici											
nella condivisione della strumentazione tecnologica											
nello svolgimento di attività lavorative in smart working											
nello svolgimento di attività lavorative al di fuori dell’ambiente domestico											

2.1.43 Che atteggiamento nota in suo/a figlio/a nei confronti della scuola a distanza?*

Rispondere solo se le seguenti condizioni sono rispettate:
La risposta era 'Sì' Alla domanda '42 [F0105]' (Durante l’emergenza Covid-19, i docenti hanno proposto delle attività a distanza?)
Scegliere la risposta appropriata per ciascun elemento:

	Per nulla - 0	1	2	3	4	5	6	7	8	9	Moltissimo - 10
Collaborativo											
Interessato											
Incuriosito											
Preoccupato											
Irrequieto											
Emotivamente volubile											

2.1.44 In concomitanza con la DaD, ha potuto notare in suo/a figlio/a cambiamenti come

Rispondere solo se le seguenti condizioni sono rispettate:
La risposta era 'Sì' Alla domanda '42 [F0105]' (Durante l’emergenza Covid-19, i docenti hanno proposto delle attività a distanza?) *e* La risposta era 'Infanzia ' *o* 'Primaria (primo ciclo 1-2) ' *o* 'Primaria (secondo ciclo 3-5) ' Alla domanda '36 [F0101]' (Livello scolare)

Scegliere una o più delle seguenti opzioni
Scegliere **tutte** le corrispondenti:

- Difficoltà di addormentamento, protratto oltre le ore 23.00
- Difficoltà nella sveglia mattutina, protratto oltre il consueto orario
- Aumento della fame
- Diminuzione della fame
- Aumento del tempo dedicato al gioco con consolle e tablet
- Maggiore iperattività
- Difficoltà di attenzione e concentrazione
- Minor coinvolgimento e motivazione nel gioco ludico-educativo
- Paure e preoccupazioni eccessive
- Maggiore inclinazione al pianto, ai capricci, alla intolleranza al no, alle attese prolungate, etc.

2.1.45 Quale azione vorrebbe che fosse migliorata dalla scuola per una DaD efficace?

Rispondere solo se le seguenti condizioni sono rispettate:
La risposta era 'Sì' Alla domanda '42 [F0105]' (Durante l'emergenza Covid-19, i docenti hanno proposto delle attività a distanza?) *e* La risposta era 'Si' Alla domanda '37 [F0102]' ((Disabilità))
Scegliere una o più delle seguenti opzioni
Scegliere **tutte** le corrispondenti:

- Supporto tecnico (preparazione del PC, attivazione applicativi, semplificazione della connessione)
- Maggior numero di ore di collegamento
- Personalizzazione delle attività didattiche
- Attività di intrattenimento (narrazione, gioco a distanza, conversazione, visione e commento filmati, etc.)
- Collegamento più frequente con la classe (per stare con i compagni)
- Attività (compiti) più adeguati
- Confronto più puntuale con la famiglia

2.1.46 Ha notato in suo/a figlio/a cambiamenti nei livelli di attenzione e concentrazione da quando ha iniziato la DaD?*

Rispondere solo se le seguenti condizioni sono rispettate:
La risposta era 'Sì' Alla domanda '42 [F0105]' (Durante l'emergenza Covid-19, i docenti hanno proposto delle attività a distanza?) *e* La risposta era 'Scuola secondaria di I grado ' *o* 'Scuola secondaria di II grado' Alla domanda '36 [F0101]' (Livello scolare) *e* La risposta era NO 'Si' Alla domanda '37 [F0102]' ((Disabilità))
Scegli **solo una** delle seguenti:

- Sì
- No

2.1.47 Quali cambiamenti ha notato? *

Rispondere solo se le seguenti condizioni sono rispettate:
La risposta era 'Sì' Alla domanda '80 [F0126]' (Ha notato in suo/a figlio/a cambiamenti nei livelli di attenzione e concentrazione da quando ha iniziato la DaD?)
Scegliere una o più delle seguenti opzioni
Scegliere **tutte** le corrispondenti:

- spesso è distratto/a e tende ad usare altri dispositivi (cellulare, tablet) in contemporanea per svolgere attività non inerenti lo studio
- è più assonnato/a nelle ore pomeridiane (forse perché va a dormire più tardi rispetto a prima)
- è più concentrato/a ed attento/a
- Altro:

2.1.48 Ha notato in suo/a figlio/a cambiamenti nel portare a termine i compiti assegnati? *

Rispondere solo se le seguenti condizioni sono rispettate:
La risposta era 'Sì' Alla domanda '42 [F0105]' (Durante l'emergenza Covid-19, i docenti hanno proposto delle attività a distanza?) *e* La risposta era 'Scuola secondaria di I grado ' *o* 'Scuola secondaria di II grado' Alla domanda '36 [F0101]' (Livello scolare) *e* La risposta era NO 'Si' Alla domanda '37 [F0102]' ((Disabilità))
Scegliere solo una delle seguenti voci
Scegli **solo una** delle seguenti:

- Si
- No
- Non saprei

2.1.49 Quali cambiamenti ha notato in suo/a figlio/a nel portare a termine i compiti assegnati? *

Rispondere solo se le seguenti condizioni sono rispettate:
La risposta era 'Sì' Alla domanda '82 [F0127]' (Ha notato in suo/a figlio/a cambiamenti nel portare a termine i compiti assegnati?)
Scegliere solo una delle seguenti voci
Scegli **solo una** delle seguenti:

- consegna i compiti a ridosso della scadenza data dal docente
- consegna i compiti in tempo, senza aspettare la scadenza

2.1.50 Ha notato in suo/a figlio/a cambiamenti dell'umore e/o del suo modo di interagire con gli altri membri della famiglia? *

Rispondere solo se le seguenti condizioni sono rispettate:
La risposta era 'Sì' Alla domanda '42 [F0105]' (Durante l'emergenza Covid-19, i docenti hanno proposto delle attività a distanza?) *e* La risposta era 'Scuola secondaria di I grado ' *o* 'Scuola secondaria di II grado' Alla domanda '36 [F0101]' (Livello scolare) *e* La risposta era NO 'Si' Alla domanda '37 [F0102]' ((Disabilità))
Scegli **solo una** delle seguenti:

- Sì
- No

2.1.51 Ho notato che

Rispondere solo se le seguenti condizioni sono rispettate:
La risposta era 'Sì' Alla domanda '84 [F0128]' (Ha notato in suo/a figlio/a cambiamenti dell'umore e/o del suo modo di interagire con gli altri membri della famiglia?   )
Scegliere una o più delle seguenti opzioni
Scegliere **tutte** le corrispondenti:

- tende a parlare di meno con i familiari
- tende a stare isolato nella sua stanza, oltre alle ore delle lezioni e a quelle dedicate allo studio
- è più nervoso
- è triste
- gli manca fare attività sportiva con gli altri
- è più tranquillo e collaborativo
- parla più volentieri con i familiari
- cerca di più il contatto fisico

2.1.52 Rispetto a questa nuova condizione, valuti i cambiamenti delle abitudini di suo/a figlio/a nello stare in contatto con i compagni di classe rispetto *

Rispondere solo se le seguenti condizioni sono rispettate:
La risposta era 'Sì' Alla domanda '42 [F0105]' (Durante l'emergenza Covid-19, i docenti hanno proposto delle attività a distanza?) *e* La risposta era 'Scuola secondaria di I grado ' *o* 'Scuola secondaria di II grado' Alla domanda '36 [F0101]' (Livello scolare) *e* La risposta era NO 'Si' Alla domanda '37 [F0102]' ((Disabilità))
Scegliere la risposta appropriata per ciascun elemento:

	Minore - 0	1	2	3	4	Invariato - 5	6	7	8	9	Maggiore - 10	Non saprei
**al desiderio di mantenersi in contatto con i propri pari**												
**all'uso del cellulare o del computer per chattare o videochiamare i compagni e/o amici di scuola, anche in orari non abituali**												
**all'auto-esclusione dal mondo esterno, isolamento e rifiuto totale per ogni forma di relazione sociale (sindrome di Hikikomori)**												

2.1.53 L'utilizzo protratto, in questo specifico periodo, di dispositivi tecnologici ha causato in suo/a figlio/a disturbi come

Rispondere solo se le seguenti condizioni sono rispettate:
La risposta era 'Sì' Alla domanda '42 [F0105]' (Durante l'emergenza Covid-19, i docenti hanno proposto delle attività a distanza?) *e* La risposta era NO 'Si' Alla domanda '37 [F0102]' ((Disabilità))
Scegliere una o più delle seguenti opzioni
Scegliere **tutte** le corrispondenti:

- Cefalea
- Disturbi muscolo scheletrici (alterazione della postura, mal di schiena, torcicollo, tic, etc.)
- Affaticamento visivo digitale (occhi arrossati, prurito, lacrimazione)
- Capogiri
- Irrequietezza
- Altro:

[la risposta a questa domanda è facoltativa]

2.1.54 Nel corso della DaD, la scuola ha richiesto ai genitori una valutazione sulle attività didattiche proposte e/o sulle soluzioni adottate? *

Rispondere solo se le seguenti condizioni sono rispettate:
La risposta era 'Sì' Alla domanda '42 [F0105]' (Durante l'emergenza Covid-19, i docenti hanno proposto delle attività a distanza?) *e* La risposta era NO 'Scuola secondaria di II grado' Alla domanda '36 [F0101]' (Livello scolare)
Scegli **solo una** delle seguenti:

- Sì
- No

2.1.55 Nel corso della DaD, la scuola ha richiesto ai genitori o a suo/a figlio/a una valutazione sulle attività didattiche proposte e/o sulle soluzioni adottate? *

Rispondere solo se le seguenti condizioni sono rispettate:
La risposta era 'Sì' Alla domanda '42 [F0105]' (Durante l'emergenza Covid-19, i docenti hanno proposto delle attività a distanza?) *e* La risposta era 'Scuola secondaria di II grado' Alla domanda '36 [F0101]' (Livello scolare) *e* La risposta era NO 'Si' Alla domanda '37 [F0102]' ((Disabilità))
Scegliere solo una delle seguenti voci
Scegli **solo una** delle seguenti:

- No
- Solo al/alla figlio/a
- Solo a genitori
- Sia al/alla figlio/a che ai genitori

2.1.56 In fase di riprogrammazione delle attività in modalità DaD, la scuola ha coinvolto gli studenti, ad esempio attraverso la consultazione dei rappresentanti di classe o d'istituto? *

Rispondere solo se le seguenti condizioni sono rispettate:
La risposta era 'Sì' Alla domanda '42 [F0105]' (Durante l'emergenza Covid-19, i docenti hanno proposto delle attività a distanza?) *e* La risposta era 'Scuola secondaria di II grado' Alla domanda '36 [F0101]' (Livello scolare) *e* La risposta era NO 'Si' Alla domanda '37 [F0102]' ((Disabilità))
Scegli **solo una** delle seguenti:

- Sì
- No

2.1.57 Dall'inizio dell'erogazione delle attività scolastiche a distanza, ad oggi, ha potuto notare in suo/a figlio/a: *

Rispondere solo se le seguenti condizioni sono rispettate:
La risposta era 'Sì' Alla domanda '42 [F0105]' (Durante l'emergenza Covid-19, i docenti hanno proposto delle attività a distanza?) *e* La risposta era 'Scuola secondaria di I grado ' *o* 'Scuola secondaria di II grado' Alla domanda '36 [F0101]' (Livello scolare) *e* La risposta era NO 'Si' Alla domanda '37 [F0102]' ((Disabilità))
Scegliere solo una delle seguenti voci
Scegli **solo una** delle seguenti:

- una regolare partecipazione alle lezioni programmate in calendario
- una irregolare/saltuaria partecipazione alle lezioni programmate in calendario
- l'abbandono della frequenza scolastica (dispersione digitale)

2.1.58 Se lei dovesse descrivere con al massimo tre parole (es. impegnativa/stimolante/pesante/divertente...) la DaD che sta frequentando suo/a figlio/a quali sceglierebbe?

Rispondere solo se le seguenti condizioni sono rispettate:
La risposta era 'Sì' Alla domanda '42 [F0105]' (Durante l'emergenza Covid-19, i docenti hanno proposto delle attività a distanza?)
Scrivere la propria risposta qui:

2.1.59 Se lei dovesse descrivere con al massimo tre parole (es. impegnativa/stimolante/pesante/divertente...) la DaD che sta frequentando suo/a figlio/a quali sceglierebbe?

Rispondere solo se le seguenti condizioni sono rispettate:
La risposta era 'Sì' Alla domanda '216 [F0405]' (Durante l'emergenza Covid-19, i docenti hanno proposto delle attività a distanza?)
Scrivere la propria risposta qui:

La ringraziamo per la collaborazione! Le risposte fornite saranno molto utili alla ricerca di soluzioni e strategie efficaci da attuare nella DaD.

Il **team di ricerca #scuolainclusivaacasa** dell'Istituto per le Tecnologie Didattiche - Consiglio Nazionale delle Ricerche

Inviare il questionario.
Grazie per aver completato il questionario.

1 Personal data section

1.1 Personal data of the respondent

1.1.1 Gender

- Female
- Male

1.1.2 Age

- <20
- 20-24
- 25-29
- 30-34
- 35-39
- 40-44
- 45-49
- 50-54
- >54

1.1.3 Nationality

<to be selected from a list>

1.1.4 Citizenship

- Italian
- First generation immigrant
- Second generation immigrant
- Other

1.1.5 Education level

- Unschooled
- Primary education
- Lower secondary education
- Upper secondary education
- Bachelor's degree
- Master's degree

1.1.6 Employment status

- Employed
- Unemployed
- Retired
- Other

1.1.7 Are you working from home?

<This question is active if the answer to the previous question was "Employed">

- Yes
- No

1.2 Personal data of the other parent

1.2.1 Age

- <20
- 20-24
- 25-29
- 30-34
- 35-39
- 40-44
- 45-49
- 50-54
- >54

1.2.2 Nationality

<to be selected from a list>

1.2.3 Citizenship

- Italian
- First generation immigrant
- Second generation immigrant
- Other

1.2.4 Education level

- Unschooled
- Primary education
- Lower secondary education
- Upper secondary education
- Bachelor's degree
- Master's degree

1.2.5 Employment status

- Employed
- Unemployed
- Retired
- Other

1.2.6 Are you working from home?

<This question is active if the answer to the previous question was "Employed">

- Yes
- No

1.3 Family unit data

1.3.1 Province of residence

<to be selected from a list>

1.3.2 Has external support been necessary for sons/daughters management?

- Yes
- No

1.3.3 Which kind of support?

<This question is active if the answer to the previous question was affirmative>

One or more answers can be selected

- Grandparents
- Baby sitter
- Relatives
- Friends
- Other (please specify):

1.3.4 Family unit members

	Number
**Adults**	
**Sons and daughters**	
**School-age sons and daughters (from preschool to upper secondary school)**	

2 Technology section

2.1.1 Do you have ICT devices (PCs, tablets, …) at home?

- Yes
- No

2.1.2 Which type of ICT devices do you have?

<This question is active if the answer to the previous question was affirmative>

More answers can be selected

- Laptop/Netbook
- Tablet
- Desktop PC
- Printer
- Other (specify):

2.1.3 How many laptops/netbooks do you have and use regularly?

<This question is active if the respondent selected "Laptop/Netbook" as an answer to the previous questions>

- 1
- 2
- 3
- 4
- 5
- 6 or more

2.1.4 How many tablets do you have and use regularly?

<This question is active if the respondent selected "Tablet" as an answer to the questions "Which type of ICT devices do you have?">

- 1
- 2
- 3
- 4
- 5
- 6 or more

2.1.5 How many desktop PCs do you have and use regularly?

<This question is active if the respondent selected "Desktop PC" as an answer to the questions "Which type of ICT devices do you have?">

- 1

- 2
- 3
- 4
- 5
- 6 or more

2.1.6 Did your son/daughter have all the necessary technological equipment at his/ her disposal to enable him/ her to participate in distance learning courses in a timely manner?

- Yes
- No

2.1.7 Has the school supported your son/daughter technologically?

<This question is active if the answer to the previous question was negative>

- No
- By providing the family with a tablet as per ministerial indications
- By providing the family with additional necessary technological devices
- By providing the family with free access to the school network for Internet connection
- By providing the family with access to the Internet through the purchase of cellular network subscriptions

2.1.8 Compared to the ICT devices in your home prior to the COVID-19 emergency, did you have to acquire other technological equipment?

- Yes
- No

2.1.9 Indicate the number of devices acquired

<This question is active if the answer to the previous question was affirmative>

	**Number**
Laptop/Netbook	
Tablet	
Desktop PC	
Printer	
Other	

2.1.10 What kind of Internet connection do you have in your home?

- DSL
- Fiber-optic Internet
- Mobile Internet connection (via router or smartphone)

2.1.11 Was it necessary to change the Internet connection type/commercial offering as a result of the emergency?

- Yes
- No

2.1.12 Please indicate your level of competence for each of the items below

Choose the appropriate answer for each item

	Null	Low	Medium	Good	High
**Use of the most common technological devices (e.g., computers, tablets)**					
**Use of the Internet and its applications (e-mail, web)**					
**Technological environments used by your son/daughter for the Emergency Remote Education (class electronic register, teaching platforms, videoconferencing and communication systems)**					

3 Data on son/daughter

(To be repeated for each of the sons/daughters in the family)

3.1.1 Gender

- Female
- Male

3.1.2 School Level

- Preschool
- Primary school (1st cycle: 1-2)
- Primary school (2nd cycle: 3-5)
- Lower Secondary school
- Upper Secondary Education

3.1.3 Please, indicate if your child presents:*

Choose the appropriate answer for each item:

	Yes	No
**Disability**		
**Learning Specific Disorders**		
**Other Special Educational Needs**		

3.1.4 What kind of school does your son/daughter attend?

<This question is active if the school level selected for the son/daughter was "Upper Secondary Education">

- Liceo
- Technical school
- Vocational Education and Training (VET)

3.1.5 Prior to the Covid-19 emergency, did the teachers use technologies for educational activities (e.g., Interactive Whiteboard, computer, tablet, Educational Robotics)?

<This question is active if the son/daughter has no disability>

- Yes

- No

3.1.6 Indicates what technologies teachers were using

<This question is active if the answer to the previous question was affirmative>
One or more answers can be selected

- IBW - Interactive Whiteboard
- Computer
- Tablet
- Educational Robotics
- Class electronic register
- Messaging /communication systems
- System for distance education

3.1.7 Prior to the Covid-19 emergency, did teachers offer distance learning activities?

- Yes
- No

3.1.8 During the Covid-19 emergency, did teachers offer any distance activities?

- Yes
- No

3.1.9 What kind of disability does your son/daughter have?

<This question is active if the answer to the previous question was affirmative and the respondent specified some kind of disability, learning specific disorder or other special educational need for the son/daughter>

- Cognitive / Intellectual
- Physical
- Sensory (visual impairment)
- Sensory (hearing impairment)
- Autism Spectrum Disorders
- Multiple disabilities

3.1.10 Before the Covid-19 emergency, by whom was your son/daughter looked after?

<This question is active if the answer to the question "During the Covid-19 emergency, did teachers offer any distance activities?" was affirmative and the respondent specified some kind of disability, learning specific disorder or other special educational need for the son/daughter>

One or more answers can be selected

- Support teacher
- Pedagogical support staff
- Class teachers

3.1.11 Hours per week devoted by the support teacher to your son/daughter

<This question is active if the answer to the previous question was "Support teacher">

<to be selected from a list of values ranging from 1 to 36>

3.1.12 Hours per week devoted by the pedagogical support staff to your son/daughter

<This question is active if the answer to the question "Before the Covid-19 emergency, by whom was your son/daughter looked after?" was "pedagogical support staff">

<to be selected from a list of values ranging from 1 to 36>

3.1.13 Hours per week devoted by the class teachers to your son/daughter

<This question is active if the answer to the question "Before the Covid-19 emergency, by whom was your son/daughter looked after?" was "Class teachers">

<to be selected from a list of values ranging from 1 to 36>

3.1.14 Prior to the Covid-19 emergency, your son/daughter was attending

<This question is active if the answer to the question "During the Covid-19 emergency, did teachers offer any distance activities?" was affirmative and the respondent specified some kind of disability, learning specific disorder or other special educational need for the son/daughter>

Choose the appropriate answer for each item:

	Never	Once a month	Once a week	Two/three times a week	Every day
An individualized pathway within the classroom but with little interaction with classmates					
An individualized pathway outside the classroom					
An individualized pathway inside the classroom with					

**frequent moments of inclusion**					

3.1.15 Does your son/daughter need assistive tools (e.g. for writing, reading, communicating)?

<This question is active if the answer to the question "During the Covid-19 emergency, did teachers offer any distance activities?" was affirmative and the respondent specified some kind of disability, learning specific disorder or other special educational need for the son/daughter>

- Yes
- No

3.1.16 Specify which

<This question is active if the answer to the previous question was affirmative>

One or more answers can be selected

- Braille bar
- Voice recognition
- Voice synthesis
- Video magnifiers
- Specific technology systems for the deaf/hard of hearing
- Communicators
- Computers
- Other (please specify):

3.1.17 Since school closure, how much time has passed for Emergency Remote Education (ERE) to start?

<This question is active if the answer to the question "During the Covid-19 emergency, did teachers offer any distance activities?" was affirmative>

- 1 week
- 2 weeks
- 3 weeks
- Other

3.1.18 How many teachers have started classes in ERE mode?

<This question is active if the answer to the question "During the Covid-19 emergency, did teachers offer any distance activities?" was affirmative>

- All of them
- Most of them
- Some of them

3.1.19 Have you been contacted by the school and informed about ERE arrangements?

<This question is active if the answer to the question "During the Covid-19 emergency, did teachers offer any distance activities?" was affirmative>

- Yes
- No

3.1.20 How were you contacted?

<This question is active if the answer to the previous question was affirmative>

One or more answers can be selected

- Telephone/video call
- Email letter from the school headmaster
- SMS
- Message on WhatsApp or other messaging system
- Other (please, specify):

3.1.21 Has the school informed you about changes in educational programming due to the new ERE mode?

<This question is active if the answer to the question "During the Covid-19 emergency, did teachers offer any distance activities?" was affirmative>

- No
- Yes, only at the beginning of the period
- Yes, periodically

3.1.22 Did the school ask you what technological equipment to connect to the Internet you had?

<This question is active if the answer to the question "During the Covid-19 emergency, did teachers offer any distance activities?" was affirmative>

- Yes
- No

3.1.23 Do the teachers offer activities in real time (e.g., through videoconferencing)?

<This question is active if the answer to the question "During the Covid-19 emergency, did teachers offer any distance activities?" was affirmative, the school level of the child is preschool, and he/she has no disability>>

- Yes
- No

3.1.24 What type of activities do they offer?

<This question is active if the answer to the previous question was affirmative>
One or more answers can be selected

- Drawing
- Songs/rhymes/poems
- Creation of artefacts
- Storytelling
- Physical activity
- Other (please specify):

3.1.25 Is the presence of an adult required?

<This question is active if the answer to the question "During the Covid-19 emergency, did teachers offer any distance activities?" was affirmative>

- Yes, to start the activity
- Yes, for the whole duration of the activity
- No

3.1.26 With the new ERE mode, has the weekly timetable of teaching activities been rescheduled?

<This question is active if the answer to the question "During the Covid-19 emergency, did teachers offer any distance activities?" was affirmative and the school level of the son/daughter is not preschool>

- Yes
- No

3.1.27 With the new ERE mode, activities take place:

<This question is active if the answer to the previous question was affirmative>

- Everyday
- Almost every day
- Two/three times a week
- Once a week

3.1.28 What tools and methods are teachers using to manage ERE?

<This question is active if the answer to the question "During the Covid-19 emergency, did teachers offer any distance activities?" was affirmative>

One or more answers can be selected

- Class electronic register
- Video conferencing systems (e.g., Skype, Zoom, Google Meet, Jitsi, BigBlueButton....)
- Instant messaging (e.g., WhatsApp, Telegram)
- Platforms (Gsuite for Education, Office 365, Moodle, WeSchool, etc.)
- Shared folders
- Apps distributed by textbook publishers
- Other:

3.1.29 Do teachers of different disciplines use the same video communication system to get in touch and carry out teaching activities with students?

<This question is active if the answer to the question "During the Covid-19 emergency, did teachers offer any distance activities?" was affirmative, the school level of the son/daughter is not preschool, and he/she has no disability>

- Yes
- No

3.1.30 In order to carry out teaching activities, teachers use:

<This question is active if the answer to the question "During the Covid-19 emergency, did teachers offer any distance activities?" was affirmative, and the school level of the son/daughter is not preschool>
One or more answers can be selected

- videoconferencing
- audio/video materials (e.g., films explaining topics, reading stories, cartoons, etc.)
- digital questionnaires (forms to be filled in directly online)
- questionnaires in "paper" format (e.g., photos or scans of documents)
- shared whiteboard (e.g., the teacher uses his/her device to draw or carry out activities as if he/she were at the blackboard or on a sheet of paper and students can intervene interactively)
- screen sharing (e.g., teacher shares screen but students can only view and not intervene interactively)
- Other (please, specify):

3.1.31 Have collaborative learning activities (group activities, projects, research, etc.) been planned in this phase?

<This question is active if the answer to the question "During the Covid-19 emergency, did teachers offer any distance activities?" was affirmative, and the school level of the son/daughter is not preschool>

- Yes

- No

3.1.32 Do the teachers ask the children to send in their homework (e.g., drawings)?

<This question is active if the answer to the question "During the Covid-19 emergency, did teachers offer any distance activities?" was affirmative, the school level of the child is preschool, and he/she has no disability>>

- Yes
- No

3.1.33 Does the school your son/daughter attends provide workshops during and outside school hours? *

<This question is active if the answer to the question "During the Covid-19 emergency, did teachers offer any distance activities?" was affirmative, the school level of the son/daughter is Secondary Upper school, and he/she has no disability>>

- Yes
- No

3.1.34 These workshops have been …

<This question is active if the answer to the previous question was affirmative>>

- Suspended
- Suspended, because they cannot be carried out at a distance
- Modified, by adapting them to the distance mode
- Retained, because they already provided for distance learning.
- Other

3.1.35 Does your son/daughter ask for support during ERE activities?

<This question is active if the answer to the question "During the Covid-19 emergency, did teachers offer any distance activities?" was affirmative, and the school level of the son/daughter is not preschool>

- No, he/she is autonomous
- Yes, he/she requires it
- Yes, but I do not have the necessary skills to help him/her

3.1.36 For which activities your son/daughter asks for support during ERE

<This question is active if the answer to the previous question was "Yes, he/she requires it" or "Yes, but I do not have the necessary skills to help him/her">

One or more answers can be selected

- to start the lesson/activity
- for the whole duration of the lesson/activity
- to manage the technological equipment
- because he/she has difficulty understanding the content
- because he/she has difficulty in following the lesson/activity in distance mode

3.1.37 Lessons in ERE mode include:

<This question is active if the answer to the question "During the Covid-19 emergency, did teachers offer any distance activities?" was affirmative, and the respondent specified some kind of disability, learning specific disorder or other special educational need for the son/daughter>>

Choose the appropriate answer for each item:

	Never	Once a week	Two/three times a week	Every day
**An individualized pathway without contacts with his/her classmates**				
**Moments of interaction with his/her classmates on educational activities**				
**Moments of interaction with his/her classmates for socialization**				

3.1.38 The lessons in ERE mode…

<This question is active if the answer to the question "During the Covid-19 emergency, did teachers offer any distance activities?" was affirmative, and the respondent specified some kind of disability, learning specific disorder or other special educational need for the son/daughter>>

- ...have fostered relations between your son/daughter and the class
- ...have made relations between your son/daughter and the class worse.
- I don't know

3.1.39 In your opinion, the lessons in ERE mode had consequences in terms of ...

<This question is active if the answer to the question "During the Covid-19 emergency, did teachers offer any distance activities?" was affirmative, and the respondent specified some kind of disability, learning specific disorder or other special educational need for the son/daughter>>

Choose the appropriate answer for each item:

	Very negative 0	1	2	3	4	5	6	7	8	9	Very positive 10
**Learning**											
**Autonomy**											
**Communication**											

3.1.40 In your opinion, the ERE:

<This question is active if the answer to the question "During the Covid-19 emergency, did teachers offer any distance activities?" was affirmative, the school level of the son/daughter is not preschool, and he/she has no disability>>

One or more answers can be selected

- is promoting the acquisition of new disciplinary content
- is promoting the knowledge of more appealing learning methods
- is promoting the acquisition of digital and technological skills
- is a good opportunity for my son/daughter to grow, also in view of new future scenarios.

3.1.41 Please give your evaluation of the effectiveness/ ineffectiveness ...

<This question is active if the answer to the question "During the Covid-19 emergency, did teachers offer any distance activities?" was affirmative>>

Choose the appropriate answer for each item:

	Not very effective 0	1	2	3	4	5	6	7	8	9	Very effective 10
The educational activities proposed in ERE mode											
School-family interaction and communication											
Interaction with other parents/class representatives											

3.1.42 How much has the new way of doing school (ERE) affected the ordinary family organisation?

<This question is active if the answer to the question "During the Covid-19 emergency, did teachers offer any distance activities?" was affirmative>>

Choose the appropriate answer for each item:

	Not al all 0	1	2	3	4	5	6	7	8	9	Very much 10
Sharing of domestic space											
Sharing technological equipment											
Carrying out working at home activities											
Carrying out work activities outside home											

3.1.43 What attitude do you notice in your son/daughter towards distance learning?

<This question is active if the answer to the question "During the Covid-19 emergency, did teachers offer any distance activities?" was affirmative>>
Choose the appropriate answer for each item:

	Not at all 0	1	2	3	4	5	6	7	8	9	Very much 10
**Collaborative**											
**Interested**											
**Curious**											
**Concerned**											
**Restless**											
**Emotionally volatile**											

3.1.44 During the ERE period, did you notice any changes in your child such as...

<This question is active if the answer to the question "During the Covid-19 emergency, did teachers offer any distance activities?" was affirmative, and the school level of the child is preschool or primary school>>

One or more answers can be selected

- Difficulty falling asleep, prolonged beyond 23.00
- Difficulty waking up in the morning, prolonged beyond usual time
- Increased hunger
- Decreased hunger
- Increased time spent playing games with consoles and tablets
- Increased hyperactivity
- Difficulty in attention and concentration
- Decreased involvement and motivation in playing educational games
- Excessive fears and worries
- Increased inclination to: crying, whims, intolerance of no, prolonged waiting, etc.

3.1.45 Which action would you like to see improved by the school for effective ERE?

<This question is active if the answer to the question "During the Covid-19 emergency, did teachers offer any distance activities?" was affirmative, and the respondent specified some kind of disability, learning specific disorder or other special educational need for the son/daughter>>

One or more answers can be selected
- Technical support (set up of the PC, activating applications, simplifying the connection)

- Increased number of connection hours
- Personalisation of teaching activities
- Entertainment activities (storytelling, remote play, conversation, watching and commenting on films, etc.)
- More frequent connection with the class (in order to stay in contact with classmates)
- More appropriate activities (tasks)
- More frequent contacts with the family

3.1.46 Have you noticed any changes in your son/daughter’s level of attention and concentration since he/she started ERE?

<This question is active if the answer to the question “During the Covid-19 emergency, did teachers offer any distance activities?” was affirmative, the school level of the son/daughter is lower or upper secondary school, and he/she has no disability>>

- Yes
- No

3.1.47 What changes have you noticed?

<This question is active if the answer to the previous question was affirmative>>

One or more answers can be selected

- is often distracted and tends to use other devices (mobile phone, tablet) at the same time to carry out non-study-related activities
- is more sleepy in the afternoon (perhaps because he/she goes to bed later than before)
- is more concentrated and attentive
- Other:

3.1.48 Have you noticed any changes in your son/daughter's ability to complete the assigned tasks?

<This question is active if the answer to the question “During the Covid-19 emergency, did teachers offer any distance activities?” was affirmative, the school level of the son/daughter is lower or upper secondary school, and he/she has no disability>>

- Yes
- No
- I don’t know

3.1.49 What changes have you noticed in your son/daughter in completing the assigned tasks?

<This question is active if the answer to the previous question was affirmative>>

- hand in assignments close to the deadline set by the teacher

- hand in assignments on time, without waiting for the deadline.

3.1.50 Have you noticed changes in your son/daughter's mood and/or the way he/she interacts with other family members?

<This question is active if the answer to the question “During the Covid-19 emergency, did teachers offer any distance activities?” was affirmative, the school level of the son/daughter is lower or upper secondary school, and he/she has no disability>>

- Yes
- No

Specifically, what changes have you noticed?

<This question is active if the answer to the previous question was affirmative>>

One or more answers can be selected

- tends to talk less with family members
- tends to stay isolated in his room, apart from lessons and study hours
- is more nervous
- is sad
- misses playing sports with others
- is quieter and more cooperative
- talks more with family members
- seeks more physical contact

3.1.51 With respect to this new condition, assess changes in your son/daughter's habits of being in contact with classmates specifically to:

<This question is active if the answer to the question “During the Covid-19 emergency, did teachers offer any distance activities?” was affirmative, the school level of the son/daughter is lower or upper secondary school, and he/she has no disability>>

Choose the appropriate answer for each item:

	Les<br>s<br>0	1	2	3	4	Unchange<br>d<br>5	6	7	8	9	Greater<br>10	I don’t know
The desire to keep in touch with peers												
The use of a cell phone or computer to chat or make												

video calls to classmates and/or friends at school, even at unusual hours												
Self-exclusion from the outside world, isolation and total rejection of all forms of social relations (Hikikomori syndrome)												

3.1.52 The prolonged use, in this specific period, of technological devices has caused in your son/daughter disorders such as

<This question is active if the answer to the question "During the Covid-19 emergency, did teachers offer any distance activities?" was affirmative, and the son/daughter has no disability>>

One or more answers can be selected

- Headache
- Musculo-skeletal disorders (altered posture, back pain, stiff neck, nervous tics, etc.)
- Digital visual fatigue (red eyes, itching, watery eyes)
- Dizziness
- Restlessness
- Other (please, specify) ……………………………………………………………….

3.1.53 During the ERE, did the school ask parents for an evaluation of the proposed teaching activities and/or the adopted solutions?

<This question is active if the answer to the question "During the Covid-19 emergency, did teachers offer any distance activities?" was affirmative, the school level of the son/daughter is not upper secondary school>>

The answer to this question is facultative.

- Yes
- No

3.1.54 During the ERE, did the school ask the parents or your son/daughter for an evaluation of the proposed teaching activities and/or the solutions adopted?

<This question is active if the answer to the question "During the Covid-19 emergency, did teachers offer any distance activities?" was affirmative, the school level of the son/daughter is upper secondary school, and he/she has no disability>>

- No
- Only to the son/daughter
- Only to the parents
- Both to the son/daughter and to the parents

3.1.55 When replanning or rescheduling ERE activities, did the school involve students, e.g., by consulting class or school representatives?

<This question is active if the answer to the question "During the Covid-19 emergency, did teachers offer any distance activities?" was affirmative, the school level of the son/daughter is upper secondary school, and he/she has no disability>>

- Yes
- No

3.1.56 Since the start of the distance learning activities to date, have you noticed in your son/daughter:

<This question is active if the answer to the question "During the Covid-19 emergency, did teachers offer any distance activities?" was affirmative, the school level of the son/daughter is lower or upper secondary school, and he/she has no disability>>

- regular attendance at scheduled lessons
- irregular/unusual attendance at scheduled lessons
- dropping out of school (digital drop out)

3.1.57 If you had to describe with three words maximum the ERE experience of your son/daughter, which ones would you choose? (e.g., challenging; stimulating; heavy; entertaining; etc.)

<This question is active if the answer to the question "During the Covid-19 emergency, did teachers offer any distance activities?" was affirmative>>

<free text area>
